# Design of the Hydrophobic Core of Self‐Assembling Peptide Fibrils for Enhanced Neural Regeneration

**DOI:** 10.1002/smsc.202500224

**Published:** 2025-09-04

**Authors:** Yu‐Liang Tsai, Primiana Cavallo, Qi Lu, Jiyao Yu, Christopher P. Ender, Julian Link, Katrin Amann‐Winkel, Kristina Endres, Christopher V. Synatschke, Torsten John

**Affiliations:** ^1^ Max Planck Institute for Polymer Research Ackermannweg 10 55128 Mainz Germany; ^2^ Institute for Physics Johannes Gutenberg University Mainz Staudingerweg 7 55128 Mainz Germany; ^3^ Department of Psychiatry and Psychotherapy University Medical Center Mainz Johannes Gutenberg University Mainz 55131 Mainz Germany; ^4^ Faculty of Computer Sciences and Microsystems Technology Kaiserslautern University of Applied Sciences Amerikastr. 1 66482 Zweibrücken Germany; ^5^ School of Science Constructor University Campus Ring 1 28759 Bremen Germany

**Keywords:** biomaterials, fibrils, hydrophobicity, molecular dynamics, self‐assembling peptides, structural analysis

## Abstract

Neurons have limited self‐repair ability, and typical treatment approaches for damaged tissue rely on surgery. Hindered by the lack of donor tissues and the complex neural environment, there is great interest in developing biomaterials to support neural regeneration. Self‐assembling peptides with fibrous structures mimicking the extracellular matrix have shown great potential as neurosupportive biomaterials. Previously, we identified peptide sequences derived from enhancing factor‐C (EF‐C) that are neurotrophic without additional supplements. Here, a library of nine EF‐C variants is designed by varying the hydrophobic core of the peptide backbone to elucidate its influence on self‐assembly and bioactivity. The physicochemical properties of these variants, including secondary structure and morphology, are thoroughly analyzed. Furthermore, molecular dynamics simulations based on AlphaFold 3 models are conducted, providing theoretical insights that explain the differential assembly and stability of EF‐C variants. Subsequently, the peptides are tested for bioactivity in a neuroblastoma cell line (SH‐SY5Y) to establish structure–property relationships. The structure‐forming EF‐C variants, particularly those featuring phenylalanine and isoleucine, are neurotrophic toward SH‐SY5Y cells, shown by enhanced ATP levels. The combination of experimental and computational methods provides a strategy for the accelerated design of neuro‐regenerative peptides.

## Graphical abstract

This study reports the design of neurosupportive peptide fibrils by systematically varying the hydrophobic core of EF‐C‐derived peptide variants. Peptides containing phenylalanine or isoleucine side chains are identified as having high aggregation propensities and performing superior in cell culture studies. The combination of biophysical assays with computational modeling and simulation can accelerate the design of self‐assembling peptide biomaterials.

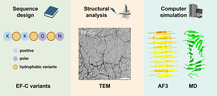

## 1 Introduction

The complex microenvironment and inhibitory factors resulting from neuroinflammation or scar formation limit the ability of neurons to repair themselves after injury.^[^ ^1^, ^2^, ^3^, ^4^ ^]^ Treatment is usually achieved by reconnecting nerve ends or integrating transplants for larger defects. However, microstructural mismatch at the injury site and limited donor tissue are major obstacles. Surgical repair of neural injuries helps patients to alleviate discomfort, but typically does not result in full neurological recovery,^[^ ^4^, ^5^, ^6^ ^]^ and more collaborative and interdisciplinary efforts in neural engineering are needed. As an alternative approach, biomaterials based on polymers, peptides, and proteins have been explored. In particular, self‐assembling peptides (SAPs) have emerged as a potential treatment for neural repair. SAPs offer a wide variety of biomimetic scaffolds that simultaneously provide different morphologies, structural stability, and biological stimuli.^[^ ^3^, ^7^, ^8^ ^]^ Peptide amphiphiles (PAs), for example, consist of a short peptide sequence conjugated to an aliphatic tail. Stupp et al. have systemically developed and thoroughly investigated their molecular design, self‐assembly dynamics, and hierarchical structure.^[^ ^9^ ^]^ PAs have been further decorated with various bioactive epitopes and growth factor mimics to improve their potential in in vitro and in vivo models of neural regeneration.^[^ ^7^, ^8^, ^10^, ^11^ ^]^ Various groups developed and investigated self‐assembling multidomain peptides, consisting of alternating hydrophilic and hydrophobic amino acids in the center (usually serine and leucine) and charged amino acids at both termini, as therapeutics for neural repair.^[^ ^12^, ^13^ ^]^ Wang et al. demonstrated that a RADA16 (sequence: RADARADARADARADA) based SAP modified with a growth factor mimic promotes angiogenesis and neurogenesis.^[^ ^14^ ^]^ Gelain and colleagues provided a comprehensive overview of this class of SAPs as well as their neural regenerative applications.^[^ ^15^ ^]^

All of the abovementioned SAPs share the incorporation of bioactive motifs into the peptide backbones to facilitate neural regeneration. In our previous work, we identified neural active peptide sequences, KIKIQI and KFKFQF, which were variants of enhancing factor‐C (EF‐C) derived from the glycoprotein GP120_417–428_ of human immunodeficiency virus.^[^ ^16^ ^]^ These sequences facilitated neuronal growth and adhesion of primary mouse neurons without the presence of epitopes or growth factor mimics.^[^ ^17^ ^]^ Our results suggested that bioactive EF‐C variants have 1) a strong tendency to form fibers, 2) an alternating pattern of hydrophobic and positively charged amino acids with a positive net charge, 3) high intermolecular *β*‐sheet contents, and 4) a larger cross‐sectional diameter in single fiber atomic force microscopy (AFM) analysis. However, the relationship between the physicochemical properties and molecular structure of the hydrophobic core of the EF‐C variants and their bioactivity has not been studied yet.

SAPs are highly versatile and easily tunable, indicating that there are multiple underlying factors, such as charge distribution and chemical nature of peptide side chains, which influence the secondary structure and supramolecular assembly.^[^ ^18^ ^]^ For example, modifying hydrophobicity and aromaticity of the SAP sequence led to different hydrogelation behavior.^[^ ^19^ ^]^ Lee et al. reported that adjusting the pattern of the SAP sequence changed the self‐assembly propensity and morphology.^[^ ^20^ ^]^ In another example, Cao and coworkers found that varying the side chain size and hydrophobicity gave rise to distinct assembled morphologies and encapsulation efficiencies of molecules.^[^ ^21^ ^]^ Yuan and colleagues found that tailoring the sequence of PAs induced different self‐assembly pathways and cell–material interactions.^[^ ^22^ ^]^ Comprehensive structural analysis is essential for understanding structure–property relationships. Characterization of SAPs typically involves molecular and microscopic analyses. At the molecular level, binding assays using dyes such as Congo Red^[^ ^23^, ^24^ ^]^ are characteristic of amyloid‐like structures with high β‐sheet content. Spectroscopy, including Fourier transform infrared spectroscopy (FTIR),^[^ ^25^, ^26^, ^27^ ^]^ and crystallography, such as X‐ray diffraction (XRD), provide additional molecular information.^[^ ^28^, ^29^ ^]^ At the microscopic level, dynamic light scattering (DLS) is a nondestructive technique to estimate the size distribution of aggregates in solution.^[^ ^18^, ^30^ ^]^ Electron microscopy, such as scanning electron microscopy and transmission electron microscopy (TEM), generates images and characterizes the morphology of SAPs.^[^ ^18^, ^30^ ^]^

**Figure 1:**
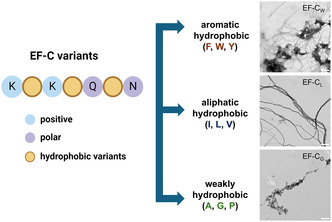
A conceptional scheme of various hydrophobic peptide motifs affecting the assembled morphology of EF‐C variants, shown in TEM images (scale bar = 500 nm).

Currently, the design of functional SAPs heavily relies on iterative experimental studies, which are not always efficient. Therefore, it is beneficial to incorporate theoretical and computational methods to reveal the formation mechanisms of SAPs and accelerate the development and prediction of functional SAPs for biomedical applications.^[^ ^31^, ^32^, ^33^ ^]^ An example of an experimental and computational study by Wang et al. showed that molecular dynamics (MD) simulations helped explain the mechanism of how the salt concentration can influence self‐assembly behavior observed in experiments.^[^ ^34^ ^]^ John and colleagues applied MD simulations to elucidate the contribution of single amino acids to the fiber formation of a sequence from islet amyloid polypeptide.^[^ ^33^ ^]^ However, predicting the accurate 3D self‐assembled structure from amino acid sequences de novo is challenging.^[^ ^35^ ^]^ In this work, we used AlphaFold 3 to predict oligomer and protofibril structures of EF‐C variants and applied MD simulations to test the stability of the predicted structures.^[^ ^36^ ^]^ We rationally designed SAPs derived from EF‐C variants with different hydrophobic motifs and analyzed their physicochemical properties. Subsequently, we compared the physicochemical analyses with theoretical predictions. Finally, a human neuroblastoma cell line (SH‐SY5Y) was used as a model to study the neural bioactivity of structure‐forming SAPs. Herein, we systematically altered the hydrophobic amino acids in the peptide backbones to gain insight into the behavior of these as neural active SAPs, as illustrated in **Figure** **1**. We aim to demonstrate that computational predictions and simulations can facilitate the design of fibril‐forming peptide sequences, which may then be used for biomaterial applications.

## 2 Results and Discussion

### 2.1 Design Strategy and Synthesis of EF‐C Variants for Enhanced Neural Regeneration

Starting from the EF‐C peptide, we systematically varied the hydrophobic core of the peptide KXKXQXN (X = hydrophobic amino acid). Previous research identified sequences consisting of KIKIQI and KFKFQF, a high propensity to form fibers, a positive net charge, and high intermolecular β‐sheet content as important features for neural repair.^[^ ^17^ ^]^ Several reports also suggested that side chain chemistry and arrangements of the peptide backbones affected the morphology, stability, and rigidity of SAPs.^[^ ^19^, ^20^, ^21^, ^37^, ^38^, ^39^, ^40^ ^]^ Inspired by these findings, nine peptide sequences were designed as variants of EF‐C. The EF‐C variants were first categorized based on their side chains into three groups: 1) EF‐C variants with rigid and aromatic side chains in the peptide backbones, which are EF‐C_F_, EF‐C_W_, and EF‐C_Y_, 2) EF‐C variants, namely EF‐C_I_, EF‐C_L_, and EF‐C_V_, with flexible and aliphatic side groups in their peptide backbones, and 3) EF‐C variants with weakly hydrophobic residues in the peptide backbones, which are EF‐C_A_, EF‐C_G_, and EF‐C_P_, respectively.

These peptides were synthesized via solid phase peptide synthesis (SPPS) using fluorenylmethyloxycarbonyl (Fmoc)‐Asn (trt) Wang resins in an automatic peptide synthesizer, and the sequences and abbreviations of the peptides are listed in **Table** **1**. All peptides were collected by reversed‐phase preparative high performance liquid chromatography (RP‐HPLC) and characterized by mass spectrometry. The purity of each peptide (≥ 90 %) was further confirmed by analytical HPLC. The corresponding spectra are provided in Figure S1, Supporting Information, and Figure S2, Supporting Information. Further characterization of leucine (EF‐C_I_) and isoleucine (EF‐C_L_) variants by ^1^H‐NMR spectroscopy is included in Figure S3, Supporting Information. To facilitate visualization of the peptide material in cell experiments, rhodamine B (RhB)‐labeled EF‐C_I_ was synthesized. Characterization of the labeling agent and product is included in Figure S4, Supporting Information, and Figure S5, Supporting Information.

**Table 1:** Overview of EF‐C variants with abbreviation, amino acid sequence, and fibril formation properties at physiological pH (7.2–7.4): conversion rate, IR peaks, XRD (calculated distance *d*), and assembled morphologies based on TEM images.

Abbreviation	Sequence	Conversion rate [%]	IR peaks 2^nd^ deriv. [cm^−1^]	XRD *d* [Å]	Morphology from TEM
EF‐C_F_	KFKFQFN	94	1626, 1694	4.7, 5.17, 5.24, 5.82	nanofiber with no or low twist
EF‐C_W_	KWKWQWN	94	1627, 1667, 1679, 1693	4.61, 4.72, 4.81, 5.06, 5.25, 8.34	nanofiber with no or low twist
EF‐C_Y_	KYKYQYN	60	1605, 1625, 1676	Xa)	no structure
EF‐C_I_	KIKIQIN	93	1620, 1658, 1678, 1690	4.66, 4.82, 5.17	twisted nanofiber
EF‐C_L_	KLKLQLN	73	1622, 1643, 1677	4.61, 4.73, 5.16, 5.26	highly twisted nanofiber
EF‐C_V_	KVKVQVN	62	1623, 16 691 688	4.64, 5.08, 5.18, 5.67, 5.83	twisted nanofiber
EF‐C_A_	KAKAQAN	27	1623, 1667, 1692	X	no structure
EF‐C_G_	KGKGQGN	19	1607, 1667	X	no structure
EF‐C_P_	KPKPQPN	35	1626, 1678	X	no structure

a) X stands for no clear signals.

### 2.2 Mechanistic Study of Self‐Assembly of EF‐C Variants

**Figure 2:**
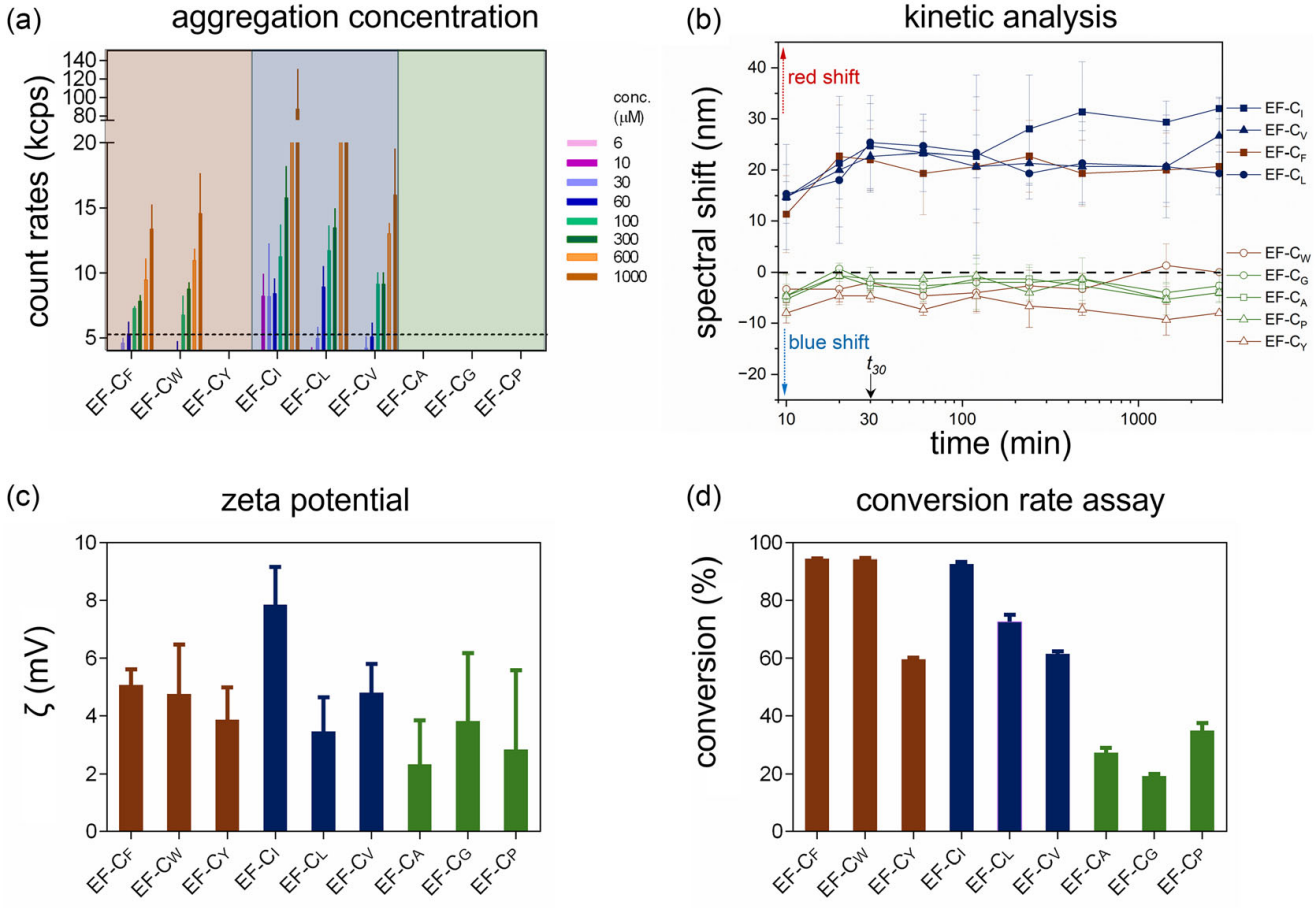
Kinetics and aggregation analysis of EF‐C variants. a) Aggregation concentrations of EF‐C variants were determined by DLS, and count rates below the dotted line (5 kcps) were considered as “no aggregation”. b) Characterization of amyloid‐like aggregation kinetics probed by Congo red assay at 1 mM peptide concentration for 2 d (measurement time points: 0 min, 10 min, 20 min, 30 min, 1 h, 2 h, 4 h, 8 h, 24 h, and 48 h). *t*_30_ stands for the time (30 min) at which assembly behavior reaches equilibrium. c) Zeta potential measurements of EF‐C variants (100 μM) after 24 h incubation displayed that the peptides have an overall positive charge. d) Conversion rates for 1 mM peptide based on a fluorescamine assay showed that EF‐C_F_, EF‐C_W_, and EF‐C_I_ had high conversation rates (x > 90%), EF‐C_L_, EF‐C_V_, and EF‐C_Y_ had moderate conversation rates (60 % < *x *< 80 %), and the remaining three variants (EF‐C_A_, EF‐C_G_, and EF‐C_P_,) had low conversation rates (x < 35%). Presented are mean values and standard deviations of the mean.

To experimentally study the self‐assembly of the nine EF‐C variants, the peptides were incubated following a uniform protocol. Each peptide was initially dissolved in dimethyl sulfoxide (DMSO) due to the high hydrophobicity of the peptides, as previously established,^[^ ^41^, ^42^ ^]^ followed by dilution in phosphate‐buffered saline to form 1 mM peptide solutions, which were statically incubated at room temperature for 24 h unless otherwise stated. To establish and verify this protocol for peptide self‐assembly, the aggregation concentration was measured by DLS. DLS is a noninvasive technique that measures the Brownian motion of molecules and correlates this motion to the size of the molecules.^[^ ^43^ ^]^ After self‐assembly of EF‐C variants for 24 h, the derived count rate of scattered light was used to determine the aggregation propensity of the peptides. In **Figure** **2** (a), five EF‐C variants, namely EF‐C_F_, EF‐C_W_, EF‐C_I_, EF‐C_L_, and EF‐C_V_, show that their aggregation concentrations are approximately or less than 100 μM, and the average size of aggregates is around 1 μm (not shown). EF‐C_I_ shows significant light scattering at the lowest concentration, thus featuring the highest aggregation propensity. The other four variants, EF‐C_Y_, EF‐C_A_, EF‐C_G_, and EF‐C_P_, demonstrated little aggregation tendency (Figure 2 (a)) with the average size of aggregates less than 20 nm (not shown). As expected, sequences with aromatic and aliphatic chains show an aggregation tendency except for EF‐C_Y_. DLS is dependent on aggregate shape and morphology and typically assumes spherical objects for accurate size determination,^[^ ^43^ ^]^ while SAPs self‐assemble into heterogeneous nonspherical aggregates. However, the method is still useful to determine aggregation tendency and is often complemented with characterization techniques such as electron microscopy.^[^ ^30^ ^]^

A Congo red assay was applied to study the kinetics of amyloid‐like fibrillar assembly based on the specific orientations of *β*‐sheet‐rich structures.^[^ ^44^ ^]^ Congo red molecules intercalate into the *β*‐sheet structure via hydrogen bonding (H‐bonding) and *π–π* stacking.^[^ ^23^, ^45^ ^]^ The interaction between Congo red molecules and *β*‐sheet‐rich structures leads to a spectral redshift in the Congo red absorption from 498 nm.^[^ ^23^, ^44^ ^]^ In Figure 2 (b), four EF‐C variants, including EF‐C_F_, EF‐C_I_, EF‐C_L_, and EF‐C_V_, show spectral redshifts at maximum absorption from 498 nm to around 520 nm. For the four variants, the spectral redshift of ≈20 nm was reached after 30 min and remained relatively stable up to 48 h, indicating that the assembly process took less than 30 min and remained in equilibrium. There were almost no spectral shifts for EF‐C_A_, EF‐C_G_, and EF‐C_P_, as expected since no aggregation was observed in DLS. Surprisingly, two sequences with aromatic side chains did not demonstrate a spectral redshift. EF‐C_W_ did not show any noticeable spectral shift and EF‐C_Y_ displayed a 10 nm blueshift, which may be attributed to the hydroxyl groups of the sequence, inducing strong H‐bonding, resulting in different electrostatic interactions between the negatively charged sulfonate groups of Congo red molecules and positively charged peptide backbones,^[^ ^45^ ^]^ while all peptides studied have an overall positive surface charge as shown in Figure 2 (c).

A conversion rate assay using fluorescamine as a probe was used to estimate the amount of peptide monomers converted into assembled structures.^[^ ^30^ ^]^ Fluorescamine is highly reactive to primary amines and the excess amount of fluorescamine is degraded within several seconds.^[^ ^46^ ^]^ EF‐C_F_, EF‐C_W_, and EF‐C_I_ demonstrate high conversion rates, while EF‐C_Y_, EF‐C_L_, and EF‐C_V_ perform rather moderately, and the monomers of EF‐C_A_, EF‐C_G_, and EF‐C_P_ barely convert into any aggregates or self‐assembled structures, following the same trends observed in the DLS study. A summary of the conversion rate assay based on fluorescamine is shown in Figure 2 (d) and included in Table 1. This approach has an intrinsic limitation in that it records the fluorescence intensity of the samples while it is unknown if amine groups are accessible in small aggregates or self‐assembled structures, leading to a potential underestimation of the fraction of aggregated molecules. In certain cases, proline and secondary amines are also reactive toward fluorescamine.^[^ ^47^ ^]^ Thus, conversion rate assays using analytical HPLC were carried out, and the results are provided in Figure S6, Supporting Information. For EF‐C_F_, EF‐C_W_, EF‐C_I_, EF‐C_L_, and EF‐C_V_, no peptide signal was observed after the samples were filtrated, suggesting that most of their monomers converted into aggregates or self‐assembled structures. On the other hand, the liquid chromatograms of EF‐C_Y_, EF‐C_A_, EF‐C_G_, and EF‐C_P_ were nearly identical before and after filtration, indicating low or no conversion. High conversion rates correlate with high aggregation tendencies, suggesting that DLS, the fluorescamine‐based conversion assay, and the analytical HPLC‐based conversion rate assay all provide similar trends for the tendency of monomers to aggregate or self‐assemble.

In summary, the protocol for peptide self‐assembly was established and validated. For the self‐assembling sequences, namely EF‐C_F_, EF‐C_W_, EF‐C_I_, EF‐C_L_, and EF‐C_V_, 1 mM of peptide was sufficient for aggregation or self‐assembly. Thus, 1 mM was selected to investigate the kinetics of each peptide, and the spectral redshift remained stable after 30 min, suggesting that 24 h of peptide incubation is sufficient to induce peptide aggregation or self‐assembly. After 24 h of incubation, all peptides exhibited a positive surface charge in buffer solution. EF‐C_F_, EF‐C_W_, EF‐C_I_, EF‐C_L_, and EF‐C_V_ showed high conversion rates. EF‐C_A_, EF‐C_G_, and EF‐C_P_ displayed only a little sign of conversion. However, EF‐C_Y_ required further analyses to confirm its aggregation tendency.

### 2.3 Morphology and Secondary Structure Analysis

**Figure 3:**
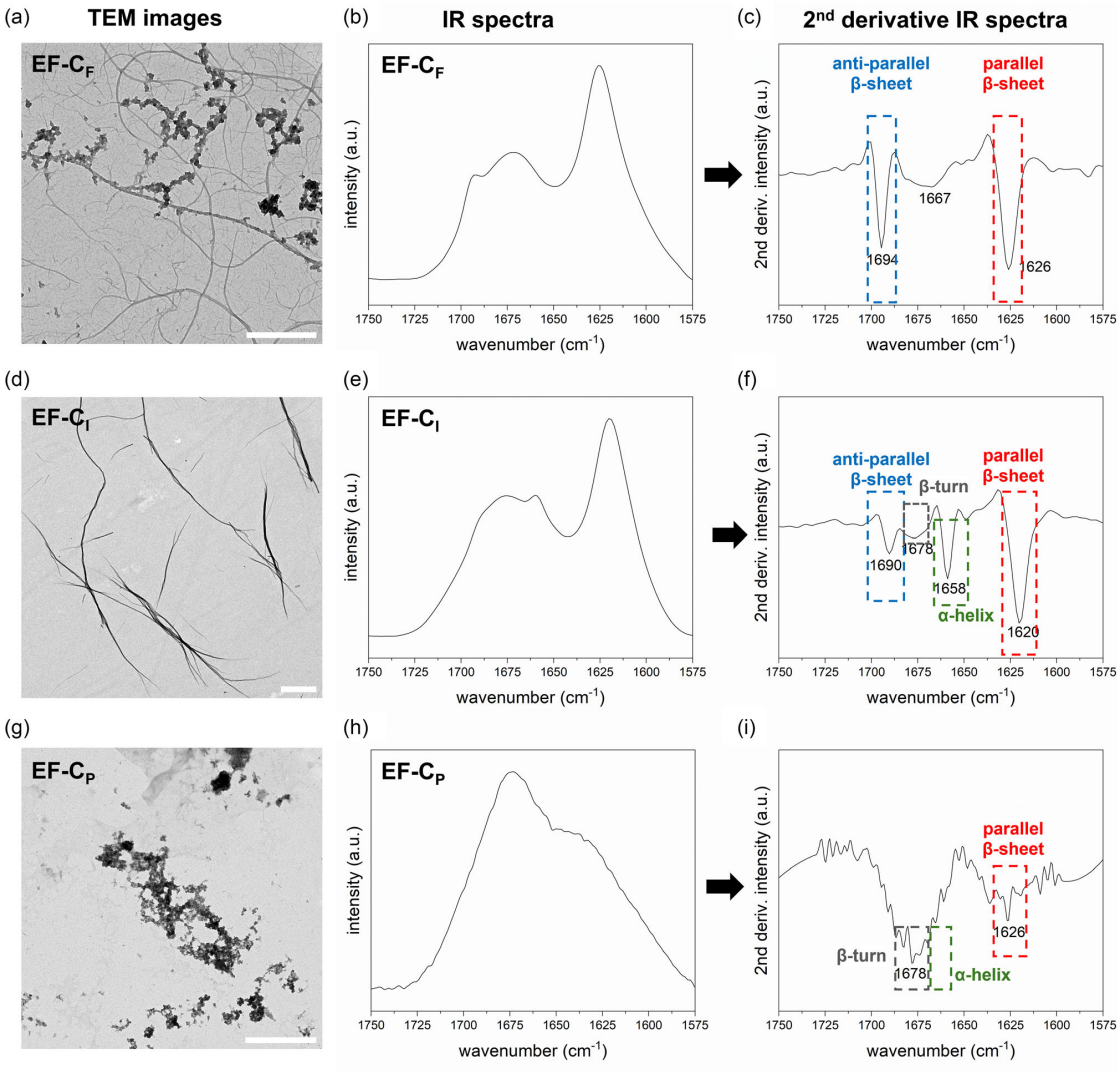
Overview of self‐assembled morphologies of EF‐C variants studied by TEM and IR. a) The representative TEM image of EF‐C_F_ reveals linear nanofibers and a small portion of aggregates (scale bar = 1 μm). b) IR absorption spectrum of EF‐C_F_. c) There are two distinct sharp peaks at 1626 cm^−1^ and 1694 cm^−1^, resulting from parallel and antiparallel β‐sheets, as shown in the 2^nd^ derivative IR absorption spectrum of EF‐C_F_. d) The representative TEM image of EF‐C_I_ shows twisted nanofibers (scale bar = 1 μm). e) IR absorption spectrum of EF‐C_I_. f) In the 2^nd^ derivative IR absorption spectrum of EF‐C_I_, there is one distinct sharp peak at 1620 cm^−1^ resulting from parallel β‐sheets, one peak attributed to α‐helix at 1658 cm^−1^, one broad peak related to β‐turn around 1678 cm^−1^, and another peak associated with antiparallel β‐sheet at 1690 cm^−1^. g) The representative TEM image of EF‐C_P_ displays aggregates only (scale bar = 1 μm). h) IR absorption spectrum of EF‐C_P_. i) In the 2^nd^ derivative IR absorption spectrum of EF‐C_P_, there is a broad band between 1640 cm^−1^ to 1680 cm^−1^, which can be assigned to β‐turn, helical, and coil structures.

With a robust protocol for the preparation of self‐assembled peptide structures, we then studied the morphologies of all EF‐C variants using TEM. A summary of morphologies is listed in Table 1, and representative TEM images are shown in **Figure** **3** (a,d,g). Additional TEM images of all variants are presented on a large scale (scale bar 2 μm) in Figure S7, Supporting Information. In the group of peptides carrying aromatic side groups, we found that EF‐C_F_ and EF‐C_W_ display fibers with no or low twist and aggregates with short and dense fibrillar networks, shown in Figure 3 (a) and Figure S7 (a,b), Supporting Information. Surprisingly, EF‐C_Y_ did not form any defined structure despite having aromatic side chains in the peptide backbones, displayed in Figure S7 (c), Supporting Information. TEM images of sequences with flexible and aliphatic side chains all exhibit long and twisted nanofibers, demonstrated in Figure 3 (d) and Figure S7 (d,e,f), Supporting Information. In contrast, in the last group of EF‐C variants with weakly hydrophobic residues, we observed many unstructured or large aggregates in Figure 3 (g) and Figure S7 (g,h,i), Supporting Information. In our aggregation studies, EF‐C_A_, EF‐C_G_, and EF‐C_P_ did not show significant aggregation tendencies or conversion rates, thus, the structures observed were most likely the result of drying artifacts.^[^ ^48^ ^]^ In Figure S8, Supporting Information, EF‐C variants that formed defined structures were further investigated with more detail to confirm the morphological observations. EF‐C_L_ fibrils are particularly twisted like twist rolls, shown in Figure S8 (d,i), Supporting Information. The assembled morphologies of the EF‐C variants were confirmed by AFM, presented in Figure S9 and S10, Supporting Information. AFM measurements revealed that the selected fibers were about 2 to 10 nm in height. We hypothesize that peptide backbones with flexible and aliphatic side groups formed longer and more twisted nanofibers because they had less steric hindrance and less directional stacking than those with aromatic groups.

After analyzing the morphologies of the EF‐C variants, we identified EF‐C_F_, EF‐C_W_, EF‐C_I_, EF‐C_L_, and EF‐C_V_ as the structure‐forming sequences, while the other four candidates are nonstructure‐forming sequences. The structural characteristics of the peptides were evaluated by IR (Figure 3, Figure S11, S12, and S13, Supporting Information) and XRD (Figure S14, Supporting Information), as summarized in Table 1. The structural information from IR primarily relies on the absorbance of the amide I band, arising mostly from the peptide C=O stretching mode, i.e., roughly from 1600 to 1700 cm^−1^ of the amide functional groups (Table S1).^[^ ^49^ ^]^ As displayed in Figure 3 (b,c,e,f) and Figure S11 and S12 (a,b,d,e,f), Supporting Information, the IR spectra of the structure‐forming sequences, including EF‐C_F_, EF‐C_W_, EF‐C_I_, EF‐C_L_, and EF‐C_V_, show a sharp peak around 1625 cm^−1^ and another peak near 1690 cm^−1^, suggesting that β‐sheets are the main secondary structure. In Figure 3 (h,i) and Figure S11 and S12 (c,g,h,i), Supporting Information, the nonfiber‐forming variants, which are EF‐C_A_, EF‐C_G_, EF‐C_P_, and EF‐C_Y_, have a dominant broad band from 1640 cm^−1^ to 1680 cm^−1^ in the IR spectra, indicating that random coils and α‐helices are the primary secondary structures. The deconvoluted IR spectra were calculated using the peaks found in the 2^nd^ derivative, provided in Figure S13, Supporting Information, and the *β*‐sheet content was calculated accordingly. The data suggest that all structure‐forming variants contained more than 68% of β‐sheets in the overall secondary structure.

XRD was used as it offers molecular packing information of amyloid‐like peptides. Typical β‐sheet patterns in protein chains assemble via H‐bonding orthogonal to the fibril direction with a distance of 4.7 Å.^[^ ^28^, ^50^ ^]^ The intermolecular packing of assembled EF‐C variants was detected by XRD, and the packing distances (d) were calculated from the XRD pattern. The XRD diffractograms are provided in Figure S14, Supporting Information, and the calculated intermolecular distances are listed in Table 1. Nonstructure‐forming sequences did not show any sharp signal that can be simply calculated from the Bragg reflection in the diffractograms (not provided). All fiber‐forming peptides had interstrand distances ranging from 4.6 to 5.8 Å featuring amyloid‐like peptides, and the differences in the packing distance between β‐sheets depended on the size of the side chain groups.^[^ ^50^ ^]^ Additional packing distances (5.2–6 Å) derived from the Bragg reflections in the XRD pattern might be attributed to intersheet distances.^[^ ^50^ ^]^ The diffraction data of EF‐C_w_ at 8.34 Å might be explained by the β‐sheets involving H‐bonding of the glutamine side chains and the secondary amine group on tryptophan side chains,^[^ ^51^ ^]^ which may be the reason why EF‐C_W_ was not reactive toward Congo red binding assay.

### 2.4 Molecular Modeling and Simulation of EF‐C Peptide Fibrils

**Figure 4:**
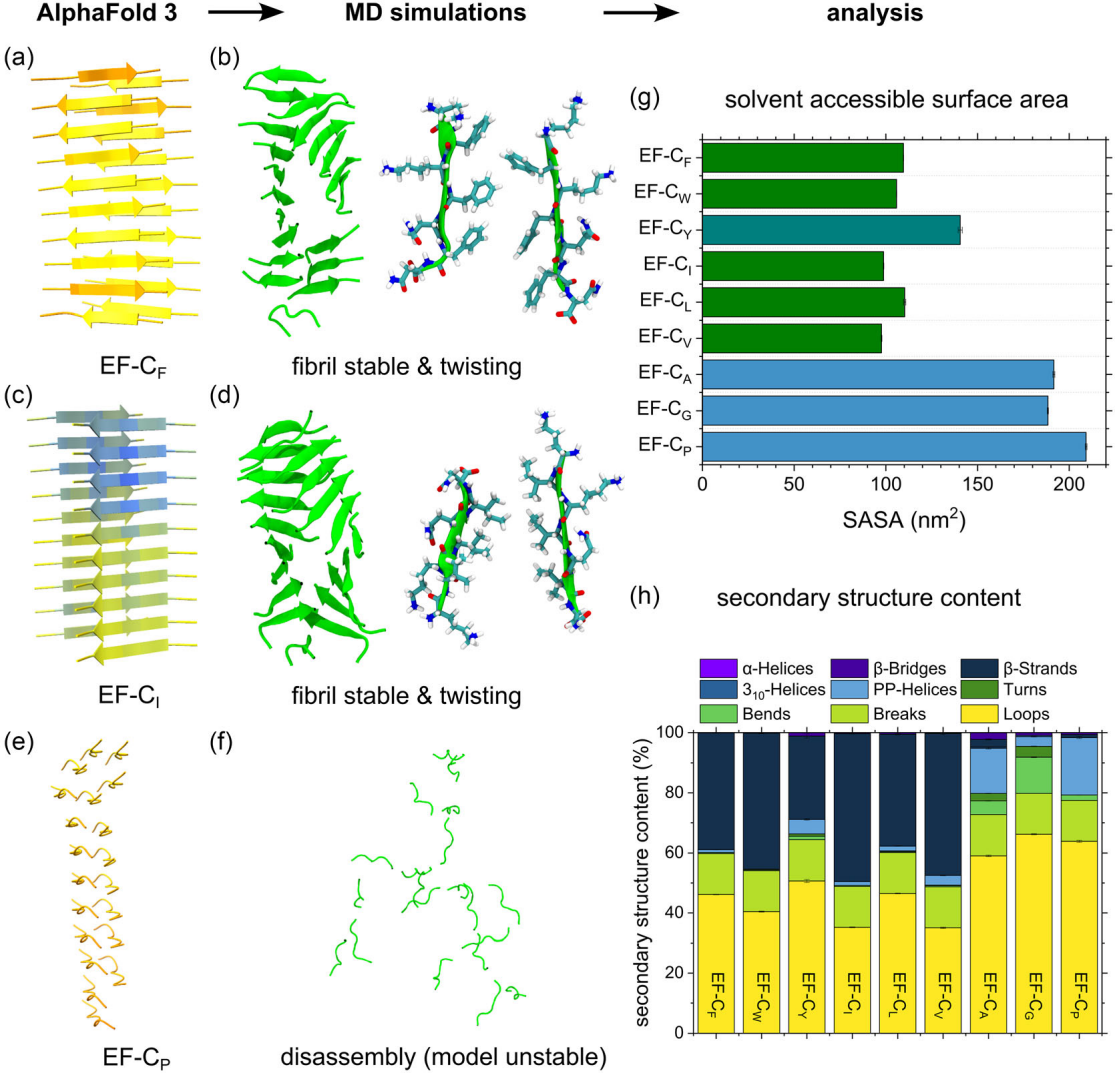
Computational AlphaFold 3 (AF3) modeling and MD simulations of EF‐C self‐assembly. a,c,e) Structures of early EF‐C protofibrils (20mers) were modeled using AF3 and then studied in all‐atom MD simulations. Confidence values of the AF3 predictions are color‐coded: high: blue, medium: yellow. b,d,f) Stable models optimized their geometry by twisting, while unstable models collapsed. Analysis of g) SASA and h) secondary structure content confirmed that stable peptide fibrils, EF‐C_F_, EF‐C_W_, EF‐C_I_, EF‐C_L_, and EF‐C_V_, had relatively high β‐sheet content, while EF‐C_A_, EF‐C_G_, and EF‐C_P_ did not have *β*‐sheet content. EF‐C_Y_ showed intermediate β‐sheet content. Secondary structures are assigned according to the DSSP classification.^[^ ^94^ ^]^

To obtain a structural model of oligomers and protofibrils of the EF‐C peptide variants, we applied AlphaFold 3 (AF3).^[^ ^52^ ^]^ AF3 enables the prediction of biomolecular interactions of complex structures using diffusion‐based machine learning. Here, we used this approach to model the structures of oligomers consisting of four peptide monomers and protofibrils consisting of twenty peptide monomers (**Figure** **4** (a,c,e) and Figure S15‐S16, Supporting Information). AF3 provided confidence values with each prediction, indicating lower scores for those structures which tend to not self‐assemble into structured fibrils. Relevant models of the oligomers and protofibrils were selected and used as starting structures for MD simulations. MD simulations sample the dynamics and stability of predicted assemblies in a solvated environment. While instable oligomer and protofibril models disassembled during MD simulation, stable peptide assemblies only presented minor structural rearrangements to optimize their geometry, shown in Figure 4 (b,d,f) and Figure S17, Supporting Information (water molecules and ions not shown for better illustration). Findings on oligomer and protofibril stability were thus indirectly related to the propensity of peptides to form peptide fibrils.^[^ ^33^ ^]^ Sequences with aliphatic side chains in their backbones (EF‐C_I_, EF‐C_L_, and EF‐C_V_) displayed slightly more twisted nanofibers compared to those with aromatic side chains in their backbones (EF‐C_F_ and EF‐C_W_), which confirmed our observation in TEM images. To quantify the observed protofibril stability, we analyzed the solvent accessible surface area (SASA), a measure for the surface area of the peptides that was accessible by water, an approach often used to analyze peptide self‐assembly.^[^ ^33^, ^36^, ^53^ ^]^ When peptide monomers assembled tightly, this area was small, while free peptide monomers had a large SASA. The nonstructural forming peptide variants, EF‐C_A_, EF‐C_G_, and EF‐C_P_, had larger SASAs compared to other EF‐C variants, displayed in Figure 4 (g) and Figure S18, Supporting Information. As an additional parameter, the secondary structure content of the peptides was determined (Figure 4 (h) and Figure S19, Supporting Information). It can be clearly seen that EF‐C_F_, EF‐C_W_, EF‐C_I_, EF‐C_L_, and EF‐C_V_ present the highest *β*–sheet content (i.e., *β*–strands and *β*–bridges in the DSSP classification), while EF‐C_A_, EF‐C_G_, and EF‐C_P_ do not have significant *β*‐sheet content.

Additional MD simulations were performed with six randomly placed peptide monomers for the fibril‐forming sequences. These simulations presented clustering of peptides in all cases (Figure S20–S22, Supporting Information) while antiparallel and parallel *β*‐sheet dimers and trimers were observed. EF‐C_W_ also formed short‐lived *β*‐sheet structures, but these were not dominant within the simulation time. A focused structure of the dimer formed during self‐assembly of EF‐C_Y_ is shown in Figure S23. Originally, EF‐C_Y_ was presumed to behave and assemble similarly to those EF‐C variants with aromatic side chains; however, it showed lower conversion rates and did not present defined structures in TEM images. With the simulation result, we propose that *π–π* interactions were the driving force to assemble EF‐C_Y_ monomers in proximity, but H‐bonding between water molecules and lysine cationic amine groups and tyrosine hydroxyl groups became the predominant interaction, leading to the collapse of the assembly. The tyrosine hydroxyl groups of the hydrophobic side chains weakened the hydrophobic core of possible assemblies. In summary, our computational results not only correlated very well with our experimental findings but also provided insights into molecular interactions, which explain the stability of each EF‐C variant. Thus, such a computational approach is suitable for screening or even predicting sequences with high propensities to form fibrils.

### 2.5 Correlation of EF‐C Fibril Assembly with SH‐SY5Y Activities

**Figure 5:**
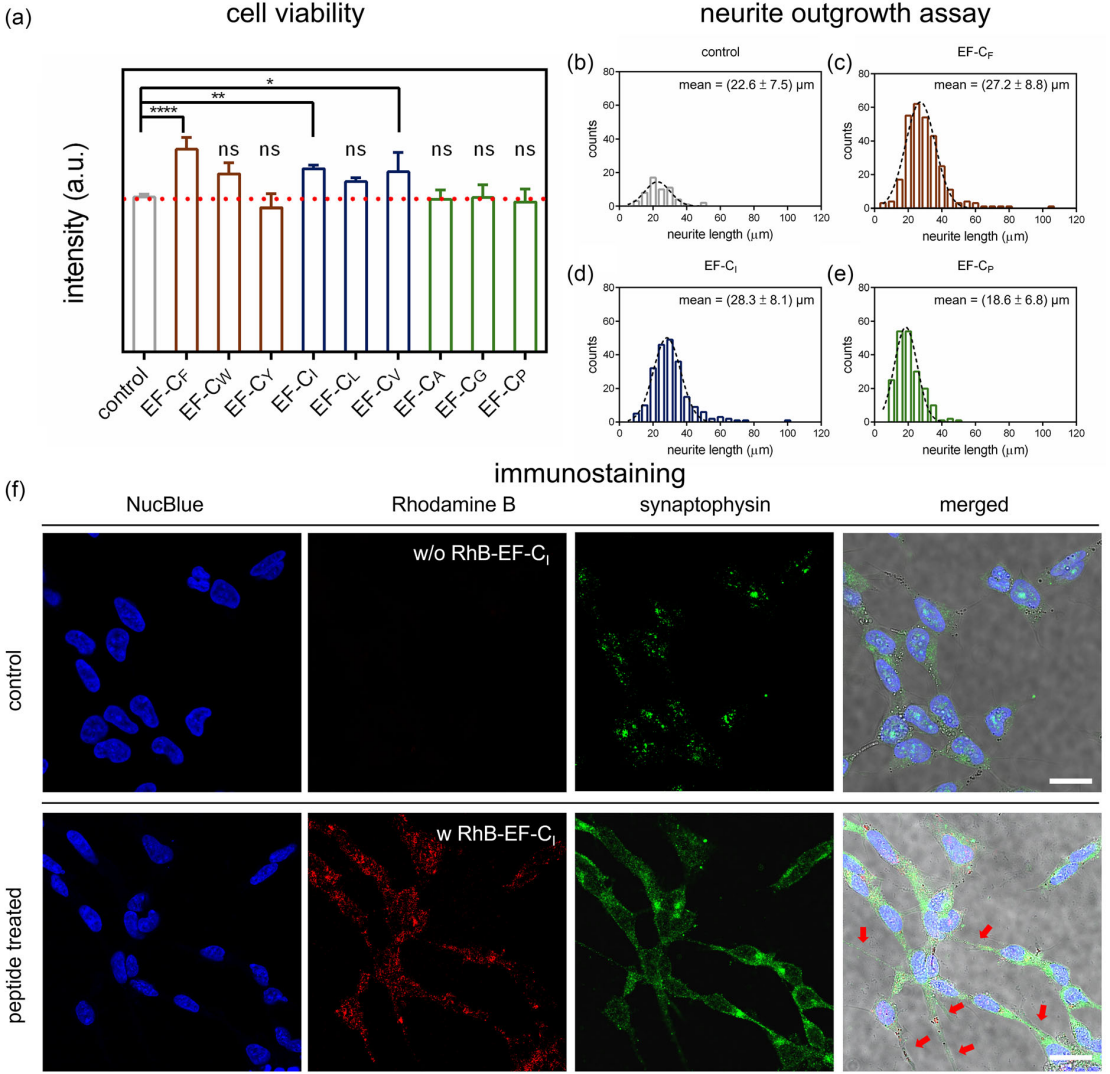
Correlations between EF‐C variants and SH‐SY5Y cell parameters. a) The graph represents cell proliferation of SH‐SY5Y cells after treatment with EF‐C variants for 24 h. The structure‐forming sequences, which are EF‐C_F_, EF‐C_W_, EF‐C_I_, EF‐C_L_, and EF‐C_V_, facilitate the proliferation of SH‐SY5Y cells, while the other sequences perform similarly to the solvent control group (p < 0.05 (*), p < 0.005 (**), p < 0.0005 (***), N = 2, n = 6, One‐way ANOVA test, Dunnett's multiple comparisons test in comparison to the solvent control). b) Neurite outgrowth assay of control group (average neurite length = 22.6 μm and SD = 7.5 μm). c) Neurite outgrowth assay of EF‐C_F_ (average neurite length = 27.2 μm and SD = 8.8 μm). d) Neurite outgrowth assay of EF‐C_I_ (average neurite length = 28.3 μm and SD = 8.1 μm). e) Neurite outgrowth assay of EF‐C_P_ (average neurite length = 18.6 μm and SD = 6.8 μm). f) The upper panel demonstrates the solvent control group with immunostained SH‐SY5Y. The lower panel shows the interaction of fluorophore‐labeled EF‐C_I_ with immunostained SH‐SY5Y. The blue channel represents the nucleus (NucBlue), the red channel is the RhB‐EF‐C_I_, the green channel is SH‐SY5Y stained with synaptophysin‐directed antibody, and the last one is the merged image with red arrows pointing at neurites (scale bar = 20 μm). The brightness and contrast of the fluorescence images of the red and green channels were slightly adjusted for better visualization, and the raw images are provided in Figure S24, Supporting Information.

After biophysical and computational characterization of the self‐assembly of the EF‐C variants, we investigated correlations between peptide structure and the characteristics of the interaction with neural cells. As reported previously, sequences of alternating hydrophobic and positively charged amino acids preserve the positive surface charges for cell adhesion.^[^ ^17^ ^]^ Based on our results, systematically switching the hydrophobic core of the peptide backbones did not significantly influence the surface charge while strongly affecting structural parameters. Building upon this, changes in bioactivity, such as proliferation and neurite outgrowth, of SH‐SY5Y should be attributed to structural variations. SH‐SY5Y cells are a secondary human neuroblastoma cell line that is widely used as a model for the analysis of neuronal functions. SH‐SY5Y cells were cultivated for 24 h before adding 50 μM of all EF‐C variants (diluted from PBS to culture medium). After incubation with EF‐C variants for 24 h, cell viability was measured using the CellTiter‐Glo assay, which is based on a luminescent signal proportional to the amount of adenosine triphosphate (ATP) present. The doubling time of SH‐SY5Y is ≈27 h;^[^ ^54^ ^]^ therefore, the results in **Figure** **5** (a) suggest that nonstructure‐forming sequences were well tolerated by the cells, but also did not provide a significant benefit over the control. On the other hand, the fiber‐forming sequences, EF‐C_F_, EF‐C_W_, EF‐C_I_, EF‐C_L_, and EF‐C_V_, induced higher ATP content in SH‐SY5Y, indicating higher cell division and metabolic activity.

In general, the differentiation of SH‐SY5Y cells requires phorbol esters, retinoic acid, or growth factors, such as nerve growth factor and brain‐derived neurotrophic factor.^[^ ^54^, ^55^, ^56^ ^]^ A neurite outgrowth assay was performed according to previous work.^[^ ^47^ ^]^ Three EF‐C variants, EF‐C_F_, EF‐C_I_, and EF‐C_P_, of each representing group, were chosen to test their potency to induce neural differentiation of SH‐SY5Y cells without adding growth factors. SH‐SY5Y cells that differentiate toward a neuroblast‐like phenotype (N‐type) are elongated and distributed randomly on a substrate, while the undifferentiated ones have an unpolarized shape and tend to grow in clusters and on top of one another.^[^ ^54^, ^57^ ^]^ Different morphologies of SH‐SY5Y cells were seen after incubation with the three peptide variants for 3 d, shown in Figure S25, Supporting Information. Cell morphologies of the control group (N = 2, n = 20, average neurite number per image = 5.1 and standard deviation (SD) = 2.0; average neurite length = 22.6 μm and SD = 7.5 μm) and EF‐C_P_ (N = 2, n = 20, average neurite number per image = 9.4 and SD = 3.6; average neurite length = 18.6 μm and SD = 6.8 μm) were less polarized and fewer neurites were observed. Contrarily, SH‐SY5Y cells became more polarized and connected with longer neurites after being treated with EF‐C_F_ (N = 2, n = 20, average neurite number per image = 13.8 and SD = 4.1; average neurite length = 27.2 μm and SD = 8.8 μm) and EF‐C_I_ (N=2, n=20, average neurite number per image = 10.4 and SD = 2.6; average neurite length = 28.3 μm and SD = 8.1 μm), displayed in Figure 5 (b‐e). We observed cells treated with structure‐forming EF‐C variants, such as EF‐C_F_ and EF‐C_I_, demonstrated slightly longer neurite outgrowth, while the nonstructure‐forming EF‐C_P_ performed similarly to the control group. We note that while the structure‐forming peptides had significant effects on cell viability, neurite outgrowth was only slightly enhanced, also in comparison to previous work that suggested similar neuroblastoma neurite lengths after incubation on flat polystyrene.^[^ ^58^ ^]^ However, only basal medium was used to culture the cells without neurotrophic molecules or growth factors. Therefore, we infer that the structure‐forming EF‐C variants cannot only boost proliferation but also induce some degree of differentiation of SH‐SY5Y cells.

Following the neurite outgrowth assay, immunochemistry was applied to verify the differentiation of SH‐SY5Y cells. Synaptophysin is a major protein of the neurotransmitter vesicle membrane, which occurs in presynaptic endings of the central and peripheral nervous systems.^[^ ^59^ ^]^ In a differentiated state, SH‐SY5Y cells express mature neuronal markers such as synaptophysin.^[^ ^54^ ^]^ On top of the immunostaining of one neuronal marker, we attempted to visualize the interaction of EF‐C variants labeled with a RhB fluorophore with neural cells. In the upper panel of Figure 5 (f), the untreated medium (DMSO only) served as the control group. The nuclei were stained with Hoechst 33342 (NucBlue). As expected, no RhB signal was observed, and only moderate expression of synaptophysin was captured based on fluorescence signals of Alexa 488 dye, and the morphology of SH‐SY5Y was less polarized. Applying the same imaging parameters, co‐assembled EF‐C_I_ and RhB‐EF‐C_I_ (*v*/*v* = 9:1) fibril assemblies, TEM images provided in Figure S26, Supporting Information, showed slight red fluorescence signals in the lower panel of Figure 5 (f). The expression of synaptophysin, based on Alexa 488 dye, was stronger. In the merged image, SH‐SY5Y cells became more polarized and neurite outgrowth, as marked with red arrows, of SH‐SY5Y cells was observed, suggesting the neural regenerative potential of the EF‐C peptide variant.

**Figure 6:**
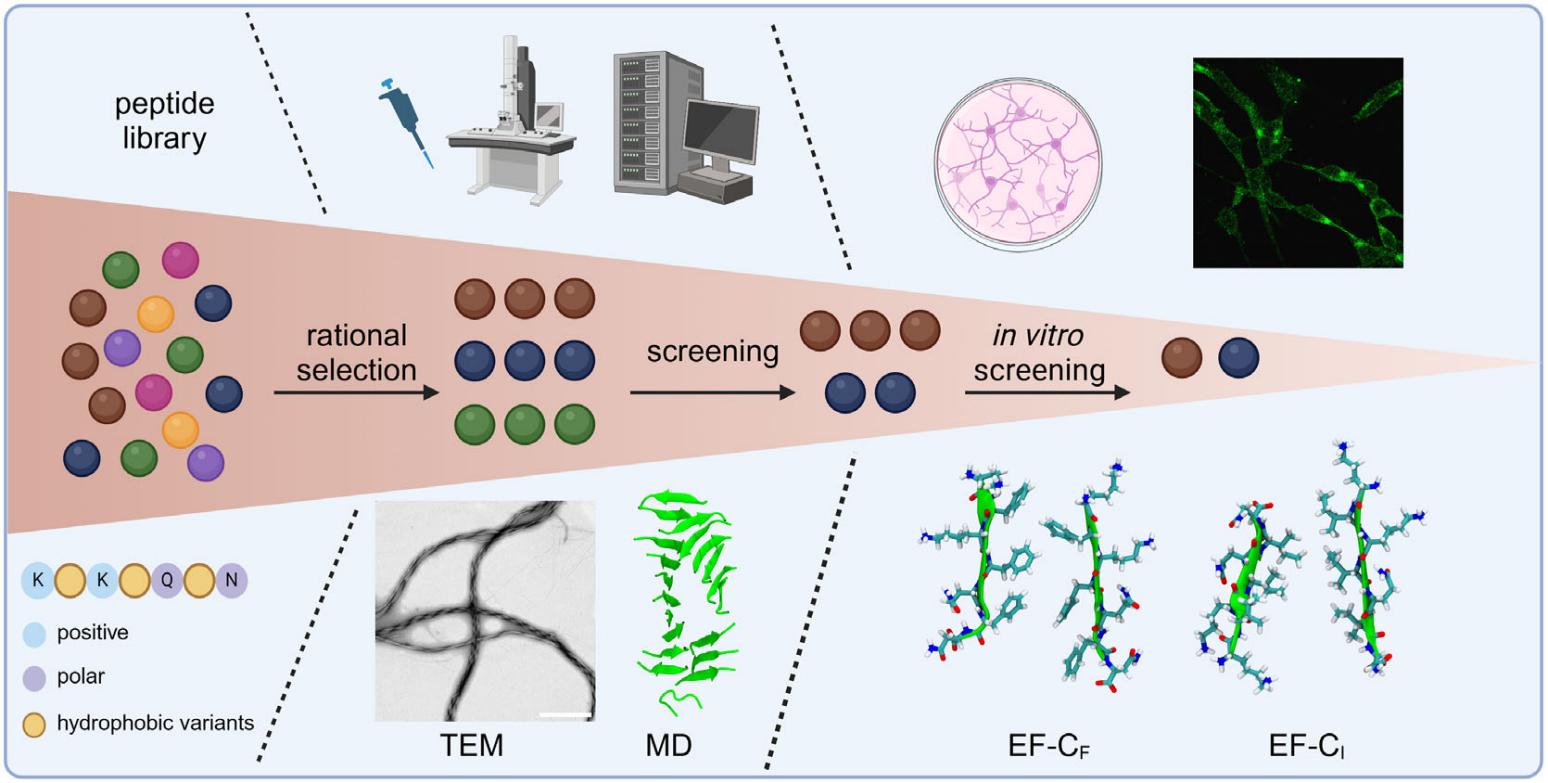
Workflow of peptide fibril materials design. Material structure–property relationships were discovered using biophysical approaches, computational modeling and simulation, and in vitro screening (image generated using Biorender).

In summary, *β*‐sheet‐rich SAPs mimic the natural extracellular matrix and provide mechanical support for cell adhesion.^[^ ^60^ ^]^ Electrostatic forces between positive surface charges of EF‐C variants and negatively charged cell membranes play an essential role in the interaction between materials and cells.^[^ ^17^ ^]^ It is still controversial, though, because some earlier research indicated that amyloid‐like structures and fragments are neurotoxic,^[^ ^61^, ^62^, ^63^ ^]^ whilst other studies demonstrated that those fibers support neural regeneration.^[^ ^64^, ^65^, ^66^, ^67^ ^]^ Based on our findings, we suggest that SH‐SY5Y viability and differentiation are facilitated by the fiber structure of EF‐C peptide variants. The mechanism for triggering SH‐SY5Y differentiation remains to be explored. In agreement with previous work, our findings demonstrate that side chain interactions of peptide monomers lead to different supramolecular structures.^[^ ^19^, ^20^, ^21^, ^39^ ^]^ Our work, summarized in **Figure** **6**, used a combination of computational modeling and simulation with biophysical experiments and in vitro screening to study the assembly and stability of these supramolecular structures. The facilitation of computational modeling and simulation can provide a strategy for materials design and the engineering of bioactive assemblies in silico. Larger data sets would be required to train neural networks for developing a quantitative model. We envision that the combination of computational approaches with biophysical characterization and biological readouts can accelerate the discovery of bioactive materials.

## 3 Conclusions

We designed a peptide library of EF‐C SAPs with varying hydrophobic cores, aiming to identify peptide fibrils with enhanced neural regeneration capacity. To achieve this, we used a systematic approach of peptide sequence variation, and each variant was characterized by biophysical imaging and spectroscopic methods. This enabled us to identify fibril‐forming sequences and their morphologies. We complemented this with computational modeling and simulation to obtain molecular‐level insight into the self‐assembly behavior. Our experimental and computational approaches correlated very well, and we thus propose that AlphaFold 3 modeling and simulations can accelerate the discovery of functional SAPs for biomaterials design. The bioactivity of each peptide variant was tested in human neuroblastoma cells, and the results suggested that the fibril material structures played a significant role in modulating cell behavior. Our study shows that the development of new peptide sequences for neural regeneration can be accelerated by combining systematic characterization of SAPs with computational modeling and simulation.

## 4 Experimental Section

4.1

4.1.1

##### Materials

Fluorenylmethyloxycarbonyl (Fmoc) protected amino acids and ethyl cyano(hydroxyimino) acetate (Oxyma Pure) were purchased from Novabiochem (Merck KGaA, Darmstadt, Germany). 6‐(Fmoc‐amino) hexanoic acid was obtained from Alfa Aesar (Thermo Fisher Scientific, Waltham, MA, USA). Fmoc‐Asn (Trt) Wang Resin was acquired from Merck KGaA (Darmstadt, Germany). Congo Red, α‐cyano‐4‐hydroxycinnamic acid (CHCA), *N,N′*‐diisopropylcarbodiimide (DIC), *N,N*‐diisopropylethylamine (DIPEA), dimethyl sulfoxide (DMSO), dimethyl sulfoxide‐d_6_ (DMSO‐d_6_), rhodamine B (RhB), triisopropylsilane (TIPS), were bought from Sigma–Aldrich (Burlington, MA, USA). *N,N*‐dimethylformamide (DMF, peptide grade), 4‐dimethylaminopyridine (DMAP), dichloromethane (DCM), fluorescamine, and 2% uranyl acetate solution were purchased from Thermo Fisher Scientific (Waltham, MA, USA). 25% ammonia solution (NH_4_OH), *N*‐(3‐dimethylaminopropyl)‐N‐ethyl carbodiimide hydrochloride salt (EDC‐HCl), *N*‐hydroxysuccinimide (NHS), piperidine (peptide grade), potassium chloride (KCl), silica gel, and trifluoroacetic acid (TFA) were obtained from Carl Roth GmbH + Co. KG (Karlsruhe, Germany). Diethyl ether and acetonitrile (ACN, HPLC grade) were acquired from Honeywell (Charlotte, NC, USA). All chemicals were used upon arrival without further purification. Ultrapure water was obtained by a Millipore purification system (MilliQ, Merck KGaA, Darmstadt, Germany).

##### Solid State Peptide Synthesis (SPPS)

All peptides were synthesized in an automated microwave peptide synthesizer (Liberty Blue, CEM Corporation, Matthews, NC, USA) from C‐ to N‐terminus by Fmoc‐SPPS method using Fmoc‐Asn (Trt) Wang Resins with a size of 100–200 mesh. DMF was used as the main washing solvent, and the deprotection solvent consisted of 20% piperidine in DMF. The activator and activator base were namely 0.5 M of DIC and 1.0 M of Oxyma in DMF. The amount of materials and reagents was prepared following the suggestion by the Liberty Blue software calculation. In brief, the resins were swollen with DMF for 30 min prior to synthesis. The procedure started with Fmoc removal, and the resins were immersed in the deprotection solvent and heated to 75 °C (155 W) for 15 s and 90 °C (30 W) for 50 s, followed by washing with DMF twice. Subsequently, amino acids were coupled from 0.2 M solutions of the respective amino acid in DMF facilitated by the addition of an activator and an activator base. The reaction was heated to 75 °C (170 W) for 15 s and 90 °C (30 W) for 110 s, followed by flushing with DMF. The deprotection solvent was added to remove the Fmoc motif after the final coupling. Afterwards, the resin beads were first flushed with DCM and immersed in a cleavage cocktail (95% TFA, 2.5% MilliQ water, 2.5% TIPS) for 2 h, followed by precipitation in cold diethyl ether and centrifugation to obtain the final precipitate. The amino acids of the peptide sequences are listed in Table 1.

##### Synthesis of N‐Hydroxysuccinimide Modified Rhodamine B (RhB‐NHS)

The synthesis of RhB‐NHS was conducted based on a previous report.^[^ ^68^ ^]^ In brief, RhB (1.0 mmol), NHS (1.1 mmol), EDC‐HCl (1.1 mmol), and DMAP (0.2 mmol) were dissolved in DCM (5 mL) in a reaction flask wrapped with aluminum foil. The resulting reaction mixture was stirred at room temperature for 4 h. The compound was purified via silica gel chromatography and was assessed by MALDI‐ToF MS on a rapifleX MALDI‐ToF/ToF (Bruker, Billerica, MA, USA) and NMR spectroscopy on a Bruker Avance 400 MHz (Bruker, Billerica, MA, USA).

##### Synthesis of RhB‐Labeled EF‐C Variants

RhB‐labeled peptides were manually synthesized on resin with a stoichiometry of 1: 2: 4 (peptide: RhB‐NHS: DIPEA) in DMF in a peptide reactor (Carl Roth GmbH + Co. KG, Karlsruhe, Germany) wrapped with aluminum foil for 4 h. Afterwards, resin beads were first rinsed with DCM and immersed in the same cleavage cocktail described previously for 2 h, followed by precipitation in cold diethyl ether and centrifugation to the final precipitate. A spacer, aminohexanoic acid, was introduced to link EF‐C_I_ and RhB‐NHS so as to diminish chemical influence on the peptide backbone.^[^ ^69^ ^]^

##### Purification and Characterization of EF‐C Variants

The peptide precipitate was dissolved in a mixture of MilliQ H_2_O and ACN and was purified by preparative RP‐HPLC (Shimadzu, Kyoto, Japan) via a C18 column (Phenomenex Gemini, 5 μm, NX‐C18, 110 Å, 150 × 30 mm, Torrance, CA, USA) with a flow rate of 25 mL min^−1^. The gradient of the solvent mixture of ACN/MilliQ with 0.1% TFA or 0.1% NH_4_OH began from 0% to 100 % ACN. Fractions of samples were collected based on the retention time monitored with an ultraviolet (UV) absorption detector at 214 nm. The purified samples were lyophilized and stored at −20 °C prior to use.

Samples were identified by MALDI‐ToF MS via the dried droplet method. Samples were mixed with a saturated solution (MilliQ H_2_O/CAN = 1:1) of the matrix CHCA. MALDI‐ToF mass spectra were recorded on a rapifleX MALDI‐ToF/ToF (Bruker, Billerica, MA, USA).

The purity of the samples was confirmed by analytical RP‐HPLC (Shimadzu, Kyoto, Japan), equipped with a C18 column (ZORRBAX Eclipse XDB‐C18, 5 μm, 70 Å, 9.4 × 250 mm, Agilent, Santa Clara, CA, USA) with a flow rate of 4 mL/min. A gradient of solvent mixture of ACN/MilliQ with 0.1% TFA or 0.1% NH_4_OH began from 5% to 80 % ACN. The samples were monitored at 214 nm UV absorption.

To distinguish between leucine (EF‐C_L_) and isoleucine (EF‐C_I_) containing variants, these two variants were dissolved in DMSO‐d_6_ with a concentration of 3 mg mL^−1^ peptide. NMR spectra were recorded on a Bruker Avance 700 MHz (Bruker, Billerica, MA, USA) at room temperature.

##### Preparation of EF‐C Self‐Assembly

Each peptide powder was dissolved in DMSO (10 mM stock solution) and further diluted into DPBS (phosphate‐buffered saline without calcium chloride and magnesium chloride, Thermo Fisher Scientific, Waltham, MA, USA) to obtain the incubation concentration of 1 mM at room temperature for 24 h, unless otherwise stated.

##### DLS

Aggregation concentrations of self‐assembling EF‐C variants were evaluated by DLS at room temperature on a Zetasizer Nano ZS (Malvern Instruments, Malvern, UK) based on published procedures.^[^ ^30^ ^]^ Peptide solutions were diluted from the 10 mM DMSO peptide stock solution into DPBS (1 mM, 600 μM, 300 μM, 100 μM, 60 μM, 30 μM, 10 μM, 6 μM) for 24 h incubation before measurement. Each measurement was performed in triplicate with 10 measurements per scan. The aggregation concentrations were plotted as count rates against peptide concentrations with Origin 8.5 (OriginLab, Northampton, MA, USA). It is worth noting that when the derived count rate was below 2 kilo counts per scan (kcps), the instrument usually displays “The sample is too dilute for measurement”. To minimize the bias, we defined no aggregation from the peptides when the derived count rate was lower than 5 kcps.

##### Congo Red Assay

Congo red assay was employed to study the formation kinetics of amyloid fibers based on a previous report.^[^ ^55^ ^]^ Congo red molecules align parallel to the fiber axis, which has a *β*‐sheet structure, and induce a redshift in the absorption maximum (498 nm). Therefore, the spectral shift was calculated only based on the absorption maximum of Congo red. The procedure is briefly described as follows: 2 μL of Congo red stock solution (500 μM) in DPBS was diluted in 43 μL PBS. 5 μL of peptide stock solution (10 mM) was added to obtain a final peptide concentration of 1 mM. The control group consisted of 5 μL DMSO, 2 μL of 500 μM Congo red and 43 μL PBS. Each mixture (15 μL) was transferred into a transparent 384‐well plate (transparent UV Star, Greiner Bio‐One, Kremsmünster, Austria), and the plate was sealed with a transparent seal (Viewseal Sealer, Greiner Bio‐One, Kremsmünster, Austria) to prevent evaporation. Absorbance shifts were recorded between 400 and 600 nm at room temperature by a microplate reader (Spark, Tecan, Männedorf, Switzerland). Analysis was performed in triplicate with 10 time points (0 min, 10 min, 20 min, 30 min, 1 h, 2 h, 4 h, 8 h, 24 h, 48 h), and the spectra were plotted with Origin 2024 (OriginLab, Northampton, MA, USA).

##### Fluorescence Based Conversion Rate (CR) Assay

The amount of peptide monomers assembled into nanostructures was assessed by a CR assay based on a previous report.^[^ ^30^ ^]^ In short, 200 μL of peptide solution (1 mM) was incubated for 24 h, and 100 μL of this solution was then centrifuged in a Vivaspin 500 centrifugal concentrator (3 kDa, Sartorius, Göttingen, Germany) to separate fibers and peptide monomers with a rate at 12 000 g for 60 min at 4 °C. Both filtrated and nonfiltrated solutions were lyophilized and dissolved in 30 μL of DMSO. 9 μL of the amine‐reactive dye fluorescamine (10 mg mL^−1^ in DMSO) was added to the filtrated and nonfiltrated ones, followed by incubation in a 384‐well plate (black UV Star, GreinerBio‐One, Kremsmünster, Austria) at room temperature for 20 min. Fluorescence was recorded on a microplate reader (Spark, Tecan, Männedorf, Switzerland) with excitation at λ = 365 nm and emission at λ = 470 nm (bandwidth = 10 nm and multiple readings per well, 3 × 3). The conversion rate was calculated using Equation (1). Each measurement was performed in triplicate and plotted with Prism 6 (GraphPad Software, San Diego, CA, USA)

(1) $$\mathrm{CR}=\left[1-\left(\frac{\mathrm{filtrated}\;\mathrm{fluorescence}\;\mathrm{intensity}}{\mathrm{nonfiltrated}\;\mathrm{fluorescence}\;\mathrm{intensity}}\right)\right]\%$$

##### Analytical HPLC‐Based CR Assay

Control groups were peptide powders dissolved in DMSO and MilliQ water at a concentration of 50 μM, and experimental groups were peptide solutions after self‐assembly (as described in “Preparation of EF‐C Self‐Assembly.”) further diluted in MilliQ water to reach the same concentration, followed by filtration through a 0.2 μm pore size filter. Analytical HPLC with the same conditions described in the previous section (“Purification and Characterization of EF‐C Variants”) was used to check whether the peptide could be detected at 214 nm UV absorption.

##### Zeta Potential (ζ)

Zeta potential measurements were conducted using a Zetasizer Nano ZS (Malvern Instruments, Malvern, UK) to determine the surface charge of aggregated peptides following a previous report.^[^ ^30^ ^]^ Peptide solutions (60 μL) were diluted in 600 μL of 1 mM KCl solution in 1 mL disposable capillary cells (DTS1060, Zetasizer Nano series, Malvern Instruments, Malvern, UK). Each measurement was performed in triplicate and plotted with Prism 6 (GraphPad Software, San Diego, CA, USA).

##### TEM

To prepare samples for TEM measurement, 5 μL of peptide solution was deposited on copper grids coated with a carbon and formvar layer (300 mesh, Plano GmbH, Wetzlar, Germany) for 10 min. The grids were stained with 2% uranyl acetate solution for 3 min, washed three times, and dried with filter paper. Measurements were performed on a Jeol 1400 electron microscope (Tokyo, Japan) with 120 kV acceleration voltage to observe the morphology of self‐assembling EF‐C variants. Images were processed with Image J.^[^ ^70^ ^]^

##### AFM

A Bruker Dimension ICON instrument (Bruker, Billerica, MA, USA) was employed to characterize the morphology of the EF‐C variants. Briefly, 10 μL of a 1 mM peptide solution was gently deposited onto freshly cleaved silicon wafers (P/Boron, <100>, Si‐Mat, Kaufering, Germany) and dried overnight in a fume hood. Imaging was performed under tapping mode using a cantilever with a resonance frequency of 300 kHz and a spring constant of 26 N m^−^^1^. The resulting AFM images were further processed and analyzed with Gwyddion 2.63 and 2.66.^[^ ^71^ ^]^

##### Attenuated Total Reflectance FTIR (ATR‐FTIR) Spectroscopy

Each peptide solution (200 μL) was lyophilized into powder before measurement. All spectra were acquired with a resolution of 4 cm^−1^ and 64 scans on a Bruker Tensor27 spectrometer (Bruker, Billerica, MA, USA) with a diamond crystal as an ATR element (PIKE MiracleTM FTIR, Madison, WI, USA). The data were plotted with Origin 8.5 (OriginLab, Northampton, MA, USA) and the second derivative of the spectra was calculated using a Savitzky–Golay filter. The *β*‐sheet content was calculated from Equation (2) based on the deconvolution area corresponding to the secondary structure and the amide I frequencies (cm^−1^) listed in Table S1.

(2) $$\text{ β}-\text{sheet }\left(\%\right)=\frac{\text{β}-\text{sheet area}}{\text{all secondary structure area}}$$

##### Powder X‐ray Diffraction (XRD)

Each peptide solution (300 μL) was lyophilized before measurement. The powders were placed on a zero reflection Si substrate (Crystal Substrates, Traverse City, MI, USA), and XRD measurements were performed in *θ*/*θ* geometry on a Rigaku SmartLab diffractometer, using a Cu K‐α anode (λ = 1.5406 Å) and HyPix‐3000 detector. The scanning angle started from 5° to 25° with a rate of 0.2° per min. The data were plotted using Origin 8.5 (OriginLab, Northampton, MA, USA), and molecular packing distances were calculated in the Match!3 (Crystal Impact, Bonn, Germany) software based on Equation (3)

(3) λ=2dsinθ

##### Modeling of Oligomer and Protofibril Structures Using AlphaFold 3

To obtain a structural model of oligomers and protofibrils of the EF‐C peptide variants, we applied AlphaFold 3 (AF3).^[^ ^52^ ^]^ The AF3 server (https://alphafoldserver.com) provided structural models of the five most likely structures, along with confidentiality scores. Relevant models of the oligomers (4mers) and protofibrils (20mers) were selected and used as starting structures for MD simulations. By default, the top structural model was chosen. When all structural models had similar confidentiality scores, the model that aligned with the other variants was chosen. In most cases, AF3 predicted 2×2 or 1×4 and 2 × 10 β‐sheets. The AF3 results are subject to the AlphaFold Server Output Terms of Use found at https://alphafoldserver.com/output‐terms. AF3 visualizations were performed with UCSF ChimeraX version 1.8, developed by the Resource for Biocomputing, Visualization, and Informatics at the University of California, San Francisco, with support from National Institutes of Health R01‐GM129325 and the Office of Cyber Infrastructure and Computational Biology, National Institute of Allergy and Infectious Diseases.^[^ ^72^ ^]^ AF3 has been used to predict the structures of peptide oligomers (4mers) and protofibrils (20mers) of nine variants.

##### Molecular Dynamics (MD) Simulations

Predicted structural models for oligomers and protofibrils from AF3 were used as starting structures for MD simulation. The structures were placed in a cubic box with box lengths of at least 5 nm (4mer, oligomer) or 7 nm (20mer, protofibril), while the minimum distance between solute and box was at least 1 nm. The peptide assemblies were solvated with explicit water using the TIP3P water model^[^ ^73^ ^]^ and sodium chloride (140 mM) was added as a physiological salt and to neutralize the overall charge of the systems. Each system was initially energy minimized using a steepest‐descent algorithm. This was followed by equilibration steps with position‐restrained peptides (k = 1000 kJ mol^−1^ nm^−2^), first in a NVT ensemble (canonical ensemble, constant number of atoms, volume, and temperature) for 100 ps and then in a NPT ensemble (isobaric‐isothermal ensemble, constant number of atoms, pressure, and temperature) for another 100 ps. Each simulation was performed in triplicate with random starting velocities following a Boltzmann distribution at 295 K. All simulations were performed for 200 ns each, while the protofibril systems that showed relatively stable structures after 200 ns were simulated for another 300 ns (total of 500 ns) to confirm structural stability of the AF3 models. The overview of simulations is included in **Table** **2**.

**Table 2:** Overview of MD simulations with simulation times and no. of replicates.

Peptide Variant	Oligomer Stability (4mer)	Protofibril Stability (20mer)	Oligomer Formation (6 monomers)
EF‐C_I_	3 × 200 ns	3 × 500 ns	3 × 500 ns
EF‐C_A_	3 × 200 ns	3 × 500 ns	/
EF‐C_G_	3 × 200 ns	3 × 200 ns	/
EF‐C_V_	3 × 200 ns	3 × 500 ns	3 × 500 ns
EF‐C_P_	3 × 200 ns	3 × 200 ns	/
EF‐C_F_	3 × 200 ns	3 × 500 ns	3 × 500 ns
EF‐C_Y_	3 × 200 ns	3 × 500 ns	3 × 500 ns
EF‐C_L_	3 × 200 ns	3 × 500 ns	3 × 500 ns
EF‐C_W_	3 × 200 ns	3 × 500 ns	3 × 500 ns

In the above studies, initial fibril structures were based on predictions from AF3. These simulations provided useful insight into the stability of oligomer and protofibril structures. To learn more about oligomer self‐assembly, systems with six randomly placed peptide monomers in a cubic box (box length 4 nm) were studied for 500 ns, except for EF‐C_A_, EF‐C_G_, and EF‐C_P_, as they all disassembled in the stability simulations and did not present characteristic fibril formation in the experiments. The monomer peptide structures were extracted from the 20mer protofibril AF3 models. As described above, these systems were also solvated, ions added, energy minimized, and equilibrated before three independent replicates were run.

GROMACS version 2021.7 was used for all MD simulations^[^ ^74^, ^75^, ^76^, ^77^, ^78^, ^79^, ^80^, ^81^ ^]^ with a time step of 2 fs and the all‐atom additive CHARMM36m force field to describe peptides, solvent, and ions.^[^ ^82^, ^83^ ^]^ Periodic boundary conditions were applied. The Verlet cutoff scheme was used for neighbor search. A single cutoff of 1.2 nm was set to calculate electrostatic and van der Waals interactions. A force switch was applied to smoothly switch the van der Waals forces to zero between 1.0 nm and 1.2 nm. Long‐range electrostatic interactions beyond the cutoff were described using the Particle–Mesh–Ewald (PME) method.^[^ ^84^ ^]^ All bonds with H‐atoms in the peptides were constrained to their equilibrium values using the LINCS algorithm;^[^ ^85^ ^]^ water molecules were constrained using the SETTLE algorithm.^[^ ^86^ ^]^ The center of mass translational velocity of the system was removed. The temperatures of “peptides” and “water and ions” were independently coupled to an external bath at 295 K using the v‐rescale thermostat with a relaxation time of 0.1 ps.^[^ ^87^ ^]^ Isotropic pressure coupling to maintain a pressure of 1 bar was achieved using the Berendsen barostat during equilibration^[^ ^88^ ^]^ and the Parrinello–Rahman barostat during production MD (relaxation time 2 ps, compressibility of 0.000045 bar^−1^).^[^ ^89^ ^]^

Analysis of MD simulations was achieved using GROMACS tools. The final 10 ns of each simulation trajectory were used for further analysis. To determine the most representative structures of all replicates for each peptide variant, cluster analysis (gmx cluster, gromos method, RMSD cutoff 0.8 nm)^[^ ^90^ ^]^ was performed and the central structure of the largest cluster was visualized using VMD 1.9.4.^[^ ^91^ ^]^ SASAs were calculated using a solvent probe radius of 0.14 nm (gmx sasa).^[^ ^92^, ^93^ ^]^ The secondary structure content of the EF‐C peptide variants was analyzed using the DSSP tool (Define Secondary Structure of Proteins, gmx dssp in GROMACS version 2024.4) for the last 10 ns of simulation time of all replicates.^[^ ^94^, ^95^ ^]^ Data were plotted with Origin 2022 (OriginLab, Northampton, MA, USA).

##### Cell Viability of Human Neuroblastoma Cells (SH‐SY5Y)

SH‐SY5Y cells (ATCC‐ CRL‐2266) were cultivated at 37 °C, 95% humidity, and 5% CO_2_ in Dulbecco's modified eagle medium (DMEM)/ Ham's F‐12 Nutrient Mixture (F‐12) (Thermo Fisher Scientific, Waltham, MA, USA) with additional 10% fetal bovine serum (FBS) (Thermo Fisher Scientific, Waltham, MA, USA) and 1% penicillin–streptomycin (PS) (Sigma–Aldrich, Burlington, MA, USA) to study cell viability proliferation after treatment with different EF‐C variants, and a control group (DMSO only). SH‐SY5Y cells were seeded at a density of 10 000 cells/well in a white half‐area 96‐well plate (Greiner Bio‐One, Kremsmünster, Austria) and incubated at 37 °C for 24 h. Peptide solutions were incubated at 1 mM for 24 h and further diluted to 50 μM in culture medium. Cells were incubated for 24 h with the respective peptide solution or solvent control (50 μL medium per well). Cell proliferation measurements were conducted using a CellTiter‐Glo Luminescence Cell Viability Assay according to the manufacturer's protocol (Promega, Madison, WI, USA). Reagent (50 μL) was added to each well and the well plate was gently shaken for 10 min in the dark at room temperature before measurement.

##### Neurite Outgrowth Assay

Each well in an 8‐well plate (glass bottom, ibidi, Gräfelfing, Germany) was first coated with a thin layer of poly‐D‐lysine hydrobromide for 1 h (concentration: 0.1 mg mL^−1^, MW = 70 000−150 000; Sigma–Aldrich, Burlington, MA, USA), rinsed with DPBS and left dry before use. SH‐SY5Y cells were seeded at a density of 80 000 cells/well in the 8‐well plate. After 24 h incubation, cells were incubated with solvent (DMSO only) or 50 μM of peptides, namely EF‐C_F_, EF‐C_I_, and EF‐C_P_, in culture medium for 3 d. The medium containing peptide solutions was refreshed on day 2. To enhance the contrast for the contour of neurites under the fluorescence microscope, cells were rinsed with PBS, followed by staining the nuclei by Hoechst 33342 dye (NucBlue, Thermo Fisher Scientific, Waltham, MA, USA) and the cell membrane according to the manufacturer's protocol (CellMask, Deep Red Plasma Membrane Stains, Invitrogen, Thermo Fisher Scientific, Waltham, MA, USA). Afterwards, cells were rinsed with PBS and fixed with 4 % *v*/*v* PFA (Sigma–Aldrich, Burlington, MA, USA) for 15 min before imaging under a BZ‐X800 microscope (Keyence, Osaka, Germany) utilizing a Plan Fluorite 40X LD PH objective lens. The neurite outgrowth was measured based on images taken at 10 random spots from two individual experiments (N = 2, n = 20), and the quantification of the neurite length was conducted on Image J and Neuron J following previous reports.^[^ ^70^, ^96^, ^97^ ^]^

##### Visualization of the Interaction of SH‐SY5Y Cells and EF‐C SAPs

SH‐SY5Y cells were seeded at the same density as described in the neurite outgrowth assay in an 8‐well plate (glass bottom, ibidi, Gräfelfing, Germany). After 24 h incubation, the cells were treated with 50 μM of a peptide solution (10% of RhB‐labeled EF‐C_I_ and 90% of unlabeled one) for another 24 h. After rinsing with PBS, cell nuclei were stained with Hoechst 33342 dye according to the manufacturer's protocol (NucBlue, Thermo Fisher Scientific, Waltham, MA, USA). For immunostaining, cells were rinsed with PBS, fixed with 4 % *v*/*v* paraformaldehyde (PFA) (Sigma‐Aldrich, Burlington, MA, USA) for 15 min, permeabilized with 0.5% *v*/*v* Triton X‐100 (Merck KGaA, Darmstadt, Germany) for 5 min, and blocked with 4 % bovine serum albumin (BSA, Roche, Basel, Switzerland) in PBS for 5 min. The primary antibody synaptophysin (SYN, rabbit anti‐SYN, Proteintech, Rosemont, IL, USA) was diluted in PBS (1:200) and added to the samples at 4 C° for 24 h. After rinsing with PBS, secondary antibody labeled with Alexa 488 (donkey anti‐rabbit IgG Alexa Fluor Plus 488, Invitrogen, Thermo Fisher Scientific, Waltham, MA, USA) in PBS (1:500) was added to samples at room temperature for 4 h before imaging.

Visualization of the cell–material interaction was conducted by using a confocal laser scanning microscope, Stellaris 8 FALCON (Leica, Wetzlar, Germany). A 405 nm excitation diode laser was implemented to monitor the nuclei staining with excitation (Ex) at 405 nm and emission (Em) at 425 nm. The immunostaining images were recorded under an Argon diode laser with Ex at 496 nm and Em at 532 nm. The fluorophore‐labeled EF‐C_I_ was observed with Ex at 546 nm and Em at 700 nm.

##### Statistical Analysis

For cell viability tests, two‐way Analysis of Variance (ANOVA) was used for comparative analysis, followed by Dunnett's multiple comparisons test. Before statistical analysis, raw data were not processed. Alpha value = 0.05 and p < 0.05 was considered statistically significant. The significance level of the calculated p values was indicated using asterisks: p > 0.05 (n.s.), p < 0.05 (*), p < 0.005 (**), and p < 0.0005 (***). For the neurite outgrowth assay, a nonlinear regression (Gaussian) was applied to calculate the mean and standard deviation of the neurite outgrowth. Prism 6 (GraphPad Software, San Diego, CA, USA) was used to process all statistical analyses. For biophysical and computational studies, details are stated with each method, while triplicate measurements were performed and mean values reported.

## Conflict of Interest

The authors declare no conflict of interest.

## Author Contributions

**Yu‐Liang Tsai**: conceptualization; methodology; validation; formal analysis; investigation; writing—original draft; writing—review and editing; visualization; project administration. **Primiana Cavallo**: formal analysis; investigation; writing—review and editing. **Qi Lu**: investigation. **Jiyao Yu**: formal analysis; investigation. **Christopher P. Ender**: formal analysis; investigation; writing—review and editing. **Julian Link**: formal analysis; investigation; writing—review and editing. **Katrin Amann‐Winkel**: methodology; formal analysis; writing—review and editing. **Kristina Endres**: methodology; formal analysis; writing—review and editing. **Christopher V. Synatschke**: resources; writing—review and editing; supervision; project administration; funding acquisition. **Torsten John**: conceptualization; methodology; formal analysis; investigation; writing—original draft; writing—review and editing; visualization; supervision; project administration.

## Supporting information

Supplementary Material

## Data Availability

Data are available in the article supplementary material and on request from the authors.
