# Supplementary material for: Design of the Hydrophobic Core of Self‐Assembling Peptide Fibrils for Enhanced Neural Regeneration
Source: Small Sci. 2025 Sep 4;5(10):2500224. doi: 10.1002/smsc.202500224 (PMC12499481; doi:10.1002/smsc.202500224)
Supplement: Supplementary file 1 — Supplementary Material [file SMSC-5-2500224-s001.zip › 3_SI_EF-C_Manuscript.pdf]

Supporting Information

Design of the Hydrophobic Core of Self-Assembling Peptide Fibrils for Enhanced Neural Regeneration

Yu-Liang Tsai, Primiana Cavallo, Qi Lu, Jiyao Yu, Christopher P. Ender, Julian Link, Katrin Amann-Winkel, Kristina Endres, Christopher V. Synatschke, and Torsten John**

Y.-L. Tsai, P. Cavallo, Q. Lu, J. Yu, C. P. Ender, J. Link, K. Amann-Winkel, C. V. Synatschke, T. John
Max Planck Institute for Polymer Research, Ackermannweg 10, 55128 Mainz, Germany
E-mail: synatschke@mpip-mainz.mpg.de, johnt@mpip-mainz.mpg.de

K. Amann-Winkel
Institute for Physics, Johannes Gutenberg University Mainz, Staudingerweg 7, 55128 Mainz, Germany

K. Endres
Department of Psychiatry and Psychotherapy, University Medical Center Mainz, Johannes Gutenberg University Mainz, 55131 Mainz, Germany

K. Endres
Faculty of Computer Sciences and Microsystems Technology, Kaiserslautern University of Applied Sciences, Amerikastr. 1, 66482 Zweibrücken, Germany

T. John
School of Science, Constructor University, Campus Ring 1, 28759 Bremen, Germany
E-mail: tjohn@constructor.university

Table of Content

Figure S1. Mass spectra of EF-C variants measured by MALDI-ToF MS.

Figure S2. Liquid chromatograms of EF-C variants.

Figure S3. NMR spectra of EF-C_I and EF-C_L.

Figure S4. Mass and ^{1}H-NMR spectra of RhB-NHS.

Figure S5. Mass spectrum and LC chromatogram of RhB-EF-C_I.

Figure S6. Conversion rate assay of self-assembled EF-C variants based on analytical liquid chromatograms measured at 214 nm absorption.

Figure S7. TEM overview images of the assembled morphologies of EF-C variants.

Figure S8. TEM detail images of the assembled morphologies of EF-C variants.

Figure S9. AFM overview images of the assembled morphologies of EF-C variants.

Figure S10. AFM detail images of the assembled morphologies of EF-C variants.

Figure S11. IR spectra of EF-C variants.

Figure S12. Second derivative IR spectra of EF-C variants.

Figure S13. Deconvoluted IR spectra of EF-C variants.

Figure S14. XRD diffractograms of self-assembled EF-C variants with assigned molecular packaging distances.

Table S1. Common secondary structures and frequencies (wavenumbers) of corresponding amide I bonds.

Figure S15. Structural models for peptide oligomers (4mers) of EF-C variants using AF3.

Figure S16. Structural models for peptide protofibrils (20mers) of EF-C variants using AF3.

Figure S17. Representative structures of peptide oligomers (4mers) and protofibrils (20mers) of EF-C variants.

Figure S18. Solvent Accessible Surface Areas (SASAs) of EF-C oligomers (4mer) and protofibrils (20mers).

Figure S19. Secondary structure content of EF-C oligomers (4mer) and protofibrils (20mers).

Figure S20. Self-assembled peptide structures of fibril-forming EF-C variants.

Figure S21. Solvent Accessible Surface Areas (SASAs) of EF-C self-assembled structures with six monomers.

Figure S22. Secondary structure content of EF-C self-assembled structures with six monomers.

Figure S23. Structural representation of dimer of EF-C_Y (KYKYQYN) formed in simulation.
Figure S24. Raw data of fluorescence images of immunostaining in Figure 5 (c).
Figure S25. Examples of neurite counting traces.
Figure S26. TEM images of co-assembled EF-C_I and RhB-EF-C_I (v/v= 9:1).
Supporting References.

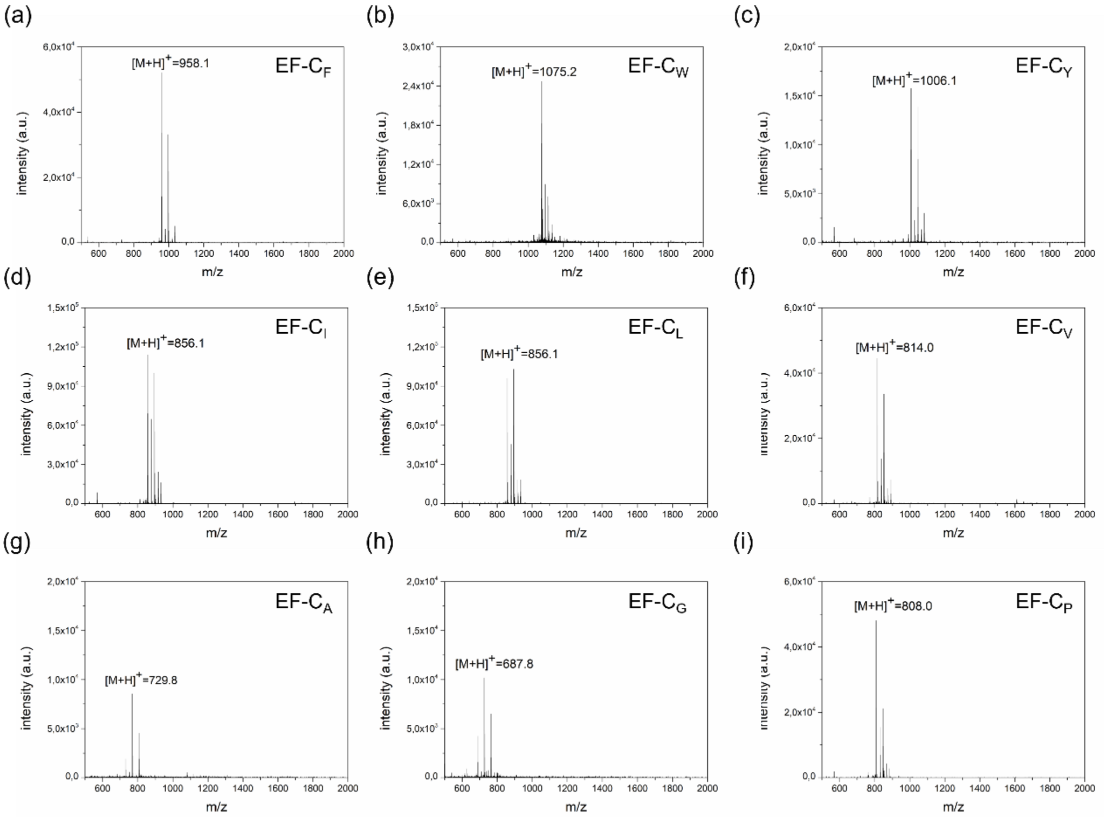

Figure S1. Mass spectra of EF-C variants measured by MALDI-ToF MS. (a) $[M+H]^+$ = 958.1 was detected for EF-C_F (theoretical MW = 958.1). (b) $[M+H]^+$ = 1075.2 was detected for EF-C_W (theoretical MW = 1075.2). (c) $[M+H]^+$ = 1006.1 was detected for EF-C_Y (theoretical MW = 1006.1). (d) $[M+H]^+$ = 856.1 was detected for EF-C_I (theoretical MW= 856.1). (e) $[M+H]^+$ = 856.1 was detected for EF-C_L (theoretical MW = 856.1). (f) $[M+H]^+$ = 814.0 was detected for EF-C_V (theoretical MW = 814.0). (g) $[M+H]^+$ = 729.8 was detected for EF-C_A (theoretical MW = 729.8). (h) $[M+H]^+$ = 687.7 was detected for EF-C_G (theoretical MW = 687.8). (i) $[M+H]^+$ = 808.0 was detected for EF-C_P (theoretical MW = 807.9).

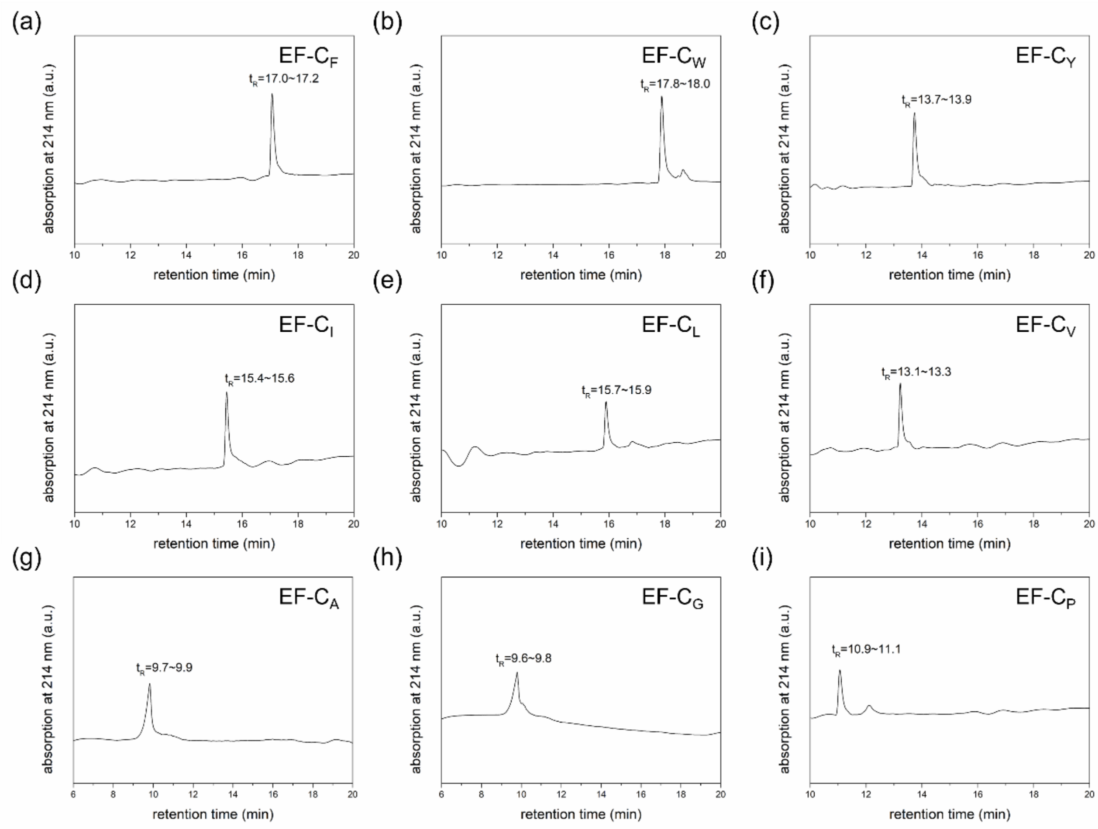

Figure S2. Liquid chromatograms of EF-C variants. (a) HPLC trace of EF-C_F in an acidic condition with a retention time (t_R) = 17.0–17.2 min. (b) HPLC trace of EF-C_W in an acidic condition with t_R = 17.8–18.0 min. (c) HPLC trace of EF-C_Y in an acidic condition with t_R = 13.7–13.9 min. (d) HPLC trace of EF-C_I in an acidic condition with t_R of 15.4–15.6 min. (e) HPLC trace of EF-C_L in an acidic condition with t_R = 15.7–15.9 min. (f) HPLC trace of EF-C_V in an acidic condition with t_R = 13.1–13.3 min. (g) HPLC trace of EF-C_A in a basic condition with a t_R = 9.7–9.9 min. (h) HPLC trace of EF-C_G in a basic condition with a t_R = 9.6–9.8 min. (i) HPLC trace of EF-C_P in an acidic condition with t_R = 10.9–11.1 min.

Given that the hydrophobic side chains of leucine (EF-C_I) and isoleucine (EF-C_L) are isomers with almost identical mass spectra and liquid chromatography profiles, distinguishing between these two peptides was complemented by additional methods. While the ^{1}H-NMR spectra showed minor differences in the aliphatic region (compare **Figure S3 (a) and (b)**), utilizing 2D proton correlation ^{1}H-NMR spectroscopy (COSY) differentiated the peptides due to the characteristic pattern of the respective amino acids, shown in **Figure S3 (c) and (d)**, thus confirming their identities.

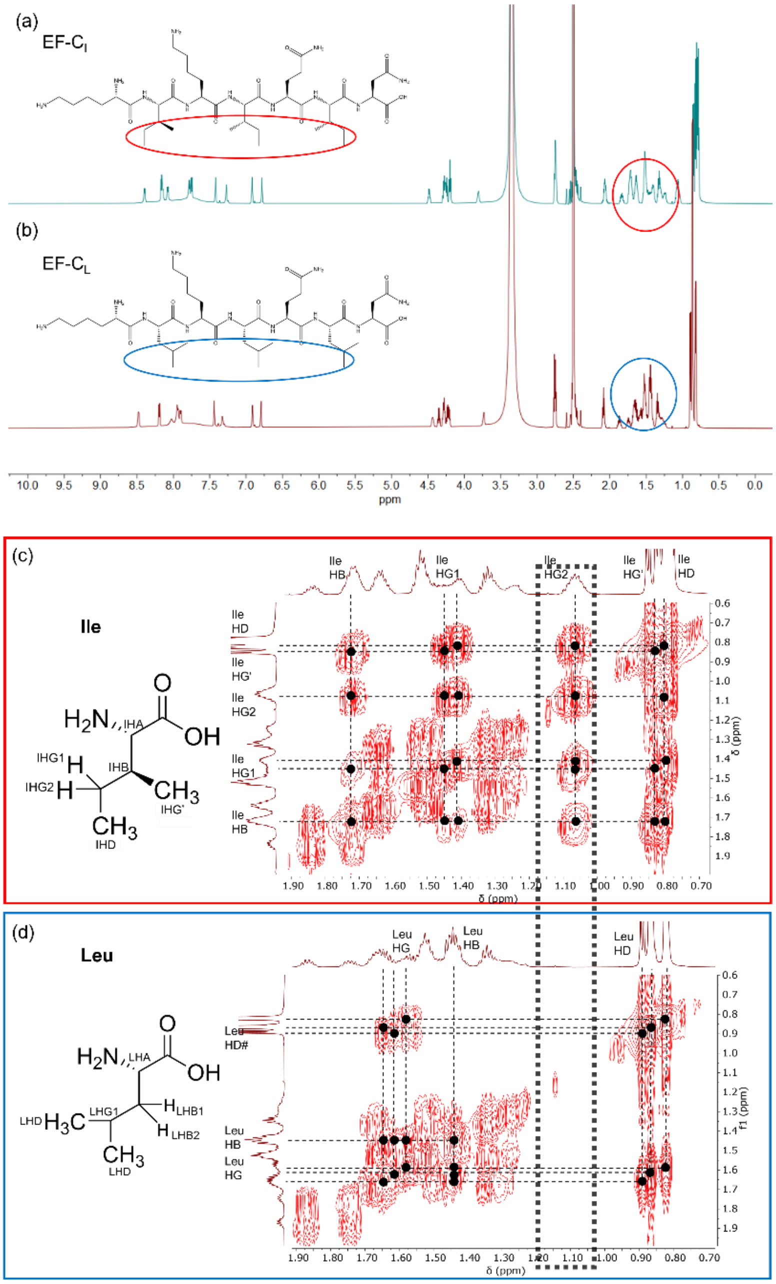

Figure S3. NMR spectra of EF-C_L and EF-C_I. (a) ^{1}H-NMR spectrum of EF-C_L. (b) ^{1}H-NMR spectrum of EF-C_I. (c) 2D-NMR correlation spectrum of EF-C_L. (d) 2D-NMR correlation spectrum of EF- C_I (DMSO-D_6, 298K, 700 MHz).

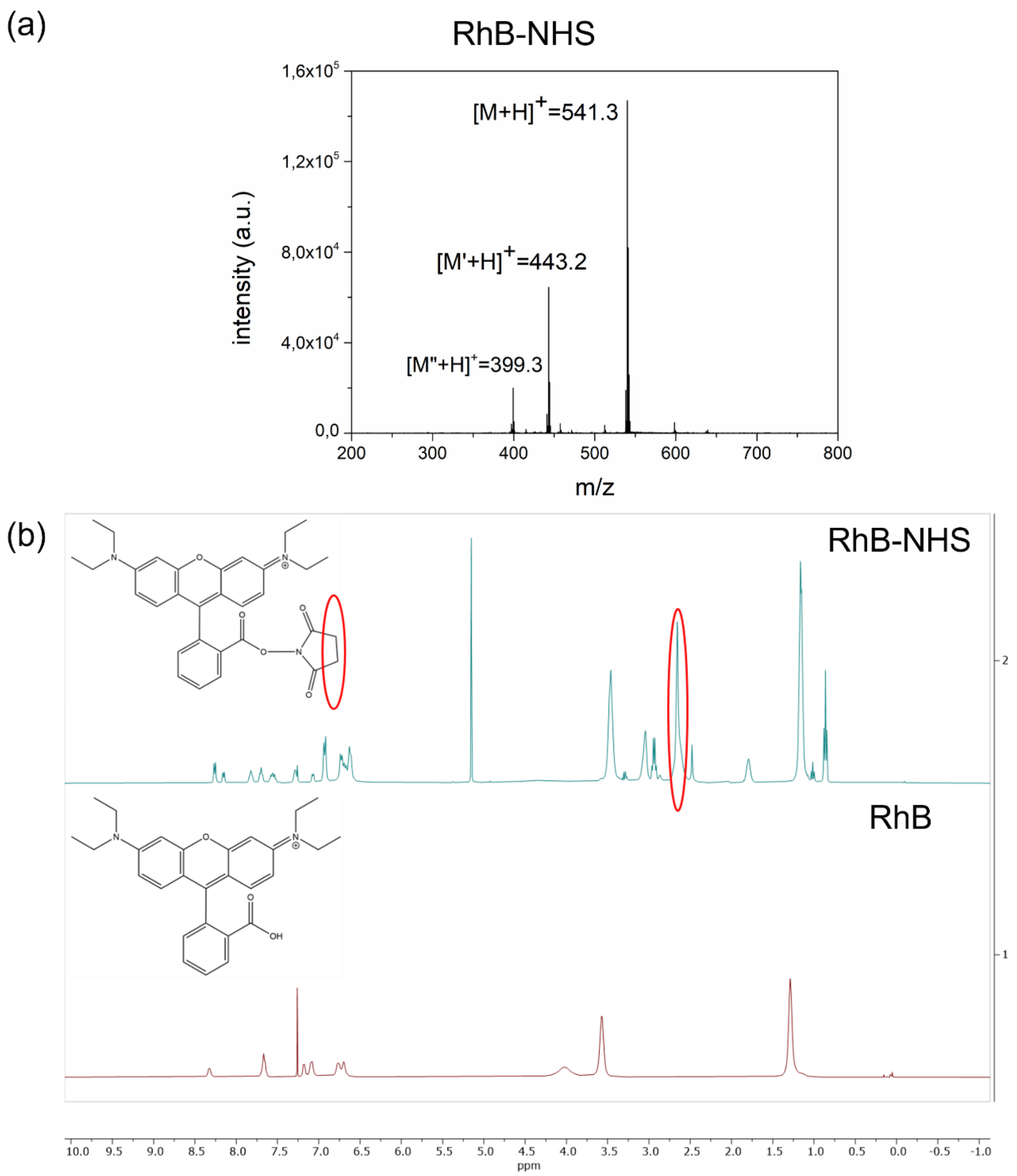

Figure S4. Mass and ^{1}H-NMR spectra of RhB-NHS. (a) Mass spectrum of RhB-NHS was measured by MALDI-ToF MS with $[M+H]^{+}$= 541.3 (theoretical MW = 540.6), while the other two signals $[M'+H]^{+}$ and $[M''+H]^{+}$ were non-fluorescent spirolactams, which could be explained by a previous report.[1] (b) ^{1}H-NMR spectra of the crude RhB-NHS product (prior to purification by silica gel chromatography) and RhB are shown. A sharp signal at 2.7 ppm is the characteristic signal for the NHS motif in the spectrum of RhB-NHS, while there is no such signal in the spectrum of RhB, indicating the successful modification of the NHS group. ($CDCl_3$, 298K, 400 MHz).

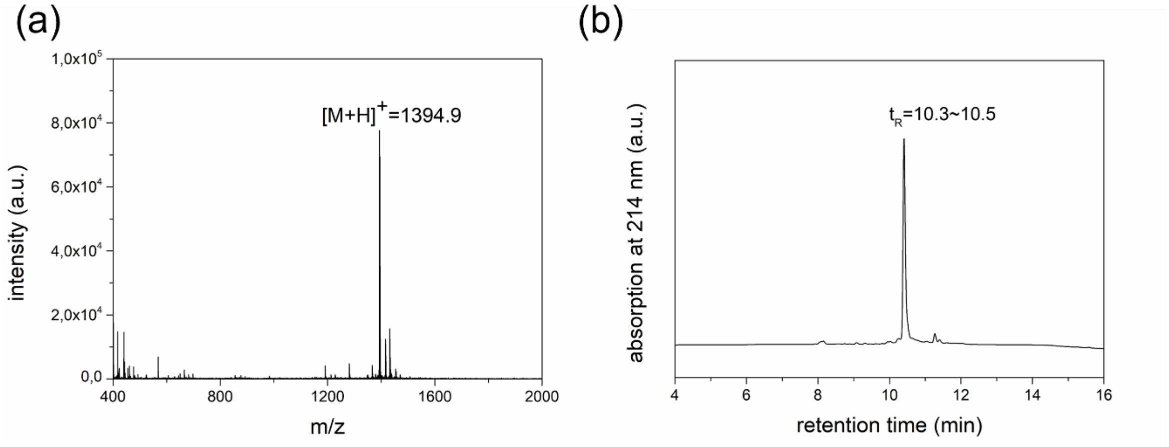

Figure S5. Mass spectrum and LC chromatogram of RhB-EF-C_I. (a) Mass spectrum of RhB-EF-C_I was measured by MALDI-TOF MS with $[M+H]^+$ = 1394.9 (theoretical MW = 1394.8). (b) HPLC trace of RhB-EF-C_I in an acidic condition with t_R = 10.3–10.5 min.

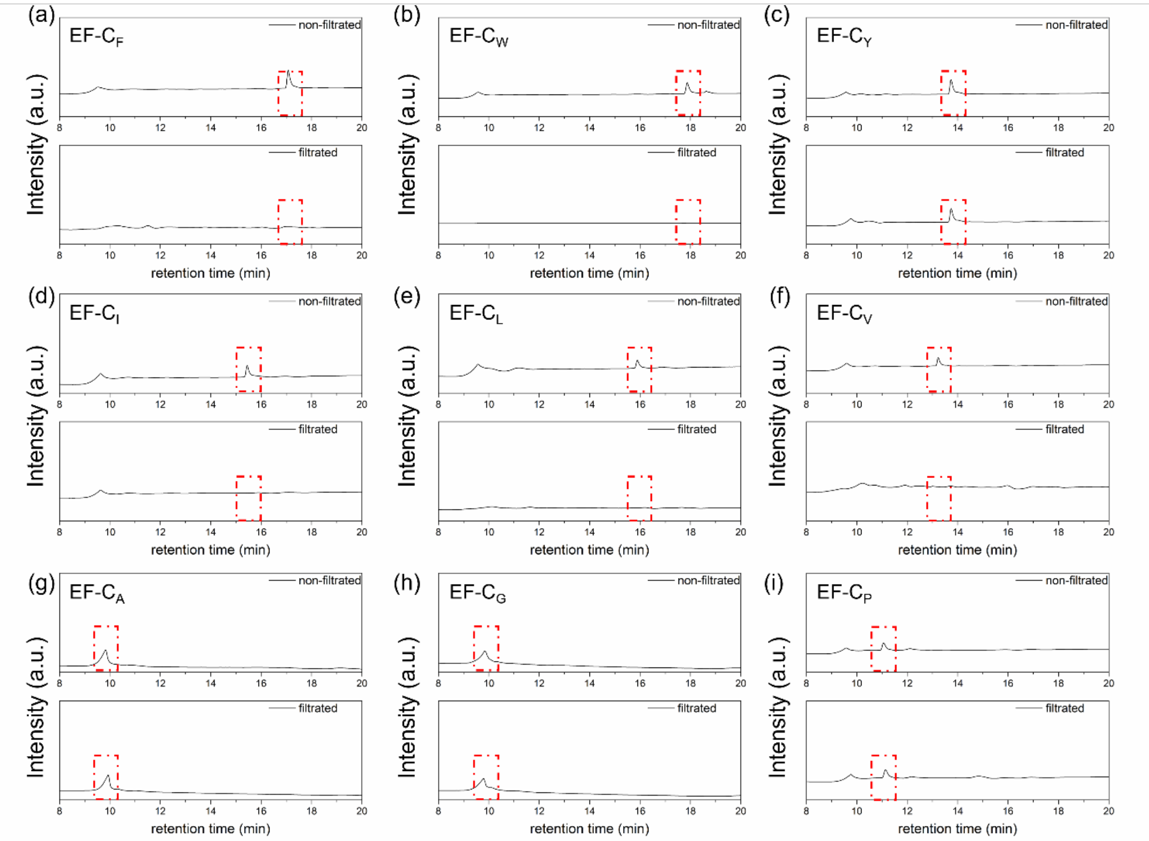

Figure S6. Conversion rate assay of self-assembled EF-C variants based on analytical liquid chromatograms measured at 214 nm absorption. (a) The upper panel of the non-filtrated sample of EF-C$_F$ shows the peptide signal, while the lower one displays no signal after filtration. (b) The upper chromograph of the non-filtered sample of EF-C$_W$ shows the peptide signal, while the lower one displays no signal after filtering. (c) Both of the panels of EF-C$_Y$ show a signal. (d) The upper panel of the non-filtrated EF-C$_I$ shows the peptide signal, and the lower panel displays no signal after filtration. (e) The upper panel of the non-filtered sample of EF-C$_L$ shows the peptide signal, while the lower one displays no signal after filtering. (f) The upper panel of the non-filtrated sample of EF-C$_V$ shows the peptide signal, while the lower one displays no signal after filtration. (g) Both panels of EF-C$_A$ show a signal with or without filtration. (h) Both panels of EF-C$_G$ display a signal with or without filtration. (i) Both panels of EF-C$_P$ show the peptide signal with or without filtration.

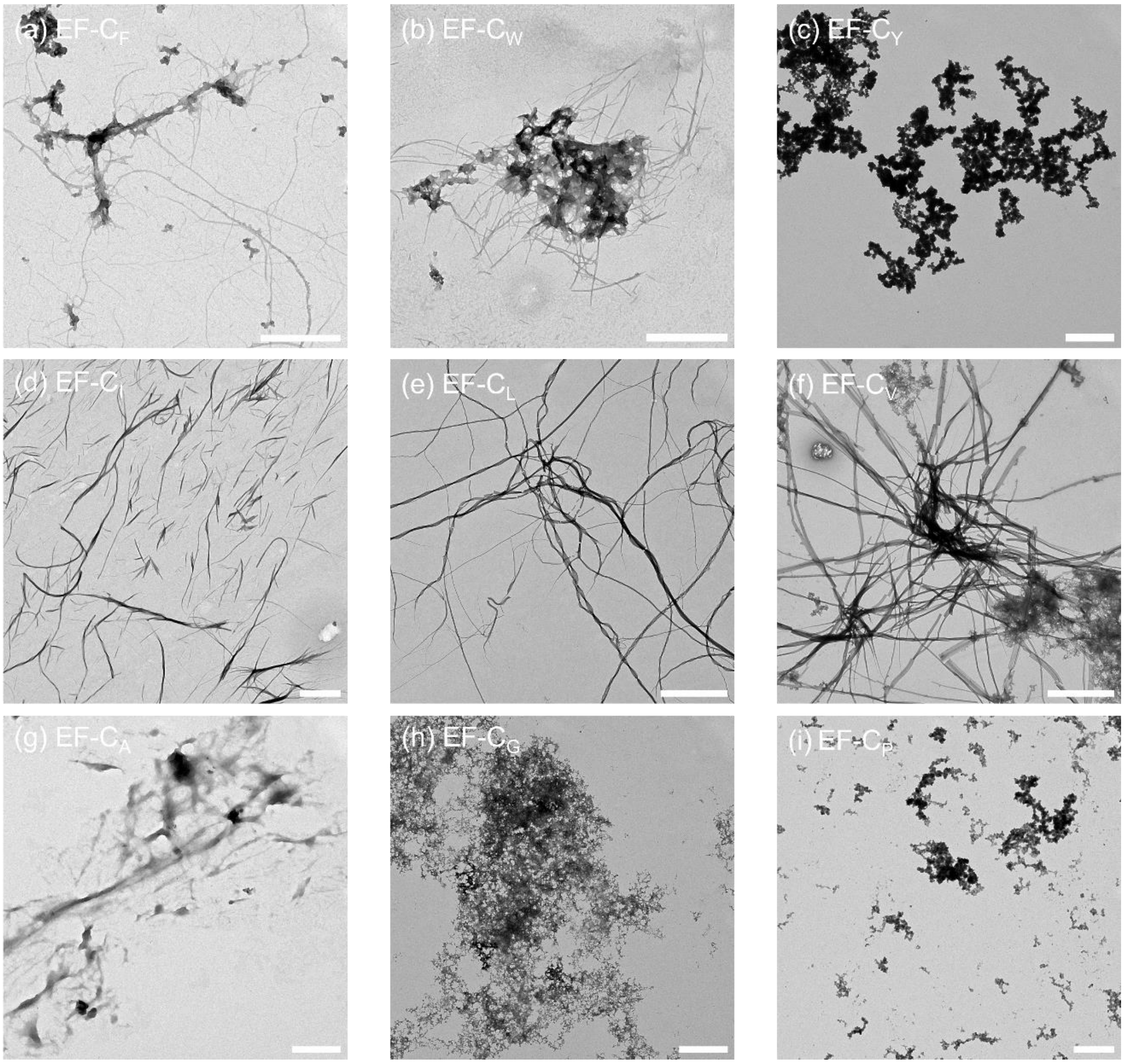

Figure S7. TEM overview images of the assembled morphologies of EF-C variants. (a) TEM image of EF-C$_F$. (b) TEM image of EF-C$_W$. (c) TEM image of EF-C$_Y$. (d) TEM image of EF-C$_I$. (e) TEM image of EF-C$_L$. (f) TEM image of EF-C$_V$. (g) TEM image of EF-C$_A$. (h) TEM image of EF-C$_G$. (i)TEM image of EF-C$_P$ (All scale bars = 2 μm).

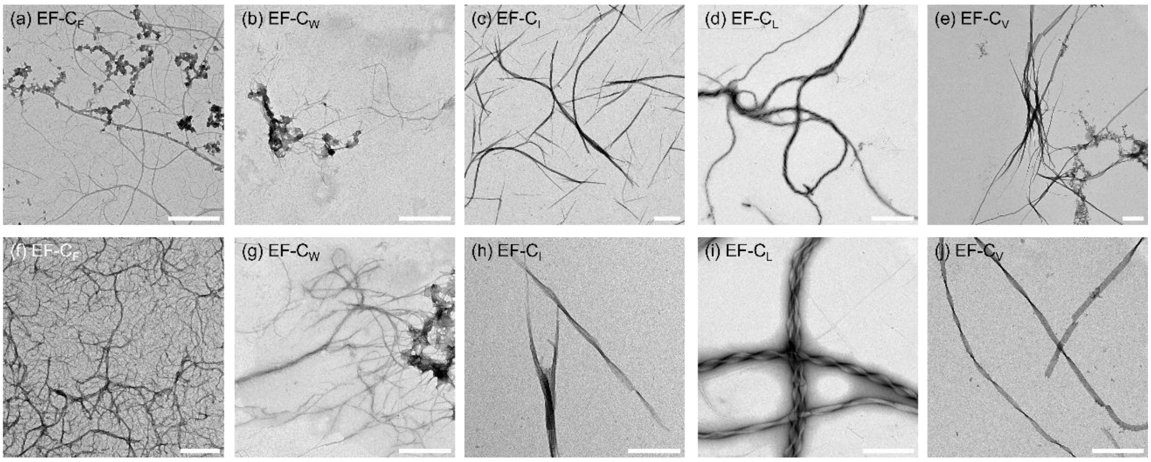

Figure S8. TEM detail images of the assembled morphologies of EF-C variants. (a, b, c, d, e) TEM images of EF-C_F, EF-C_W, EF-C_I, EF-C_L, and EF-C_V with scale bars of 1 μm. (f, h, i, j) TEM images of EF-C_F, EF-C_I, EF-C_L, and EF-C_V with scale bars of 250 nm. (g) A TEM image of EF- C_W with scale bars of 500 nm.

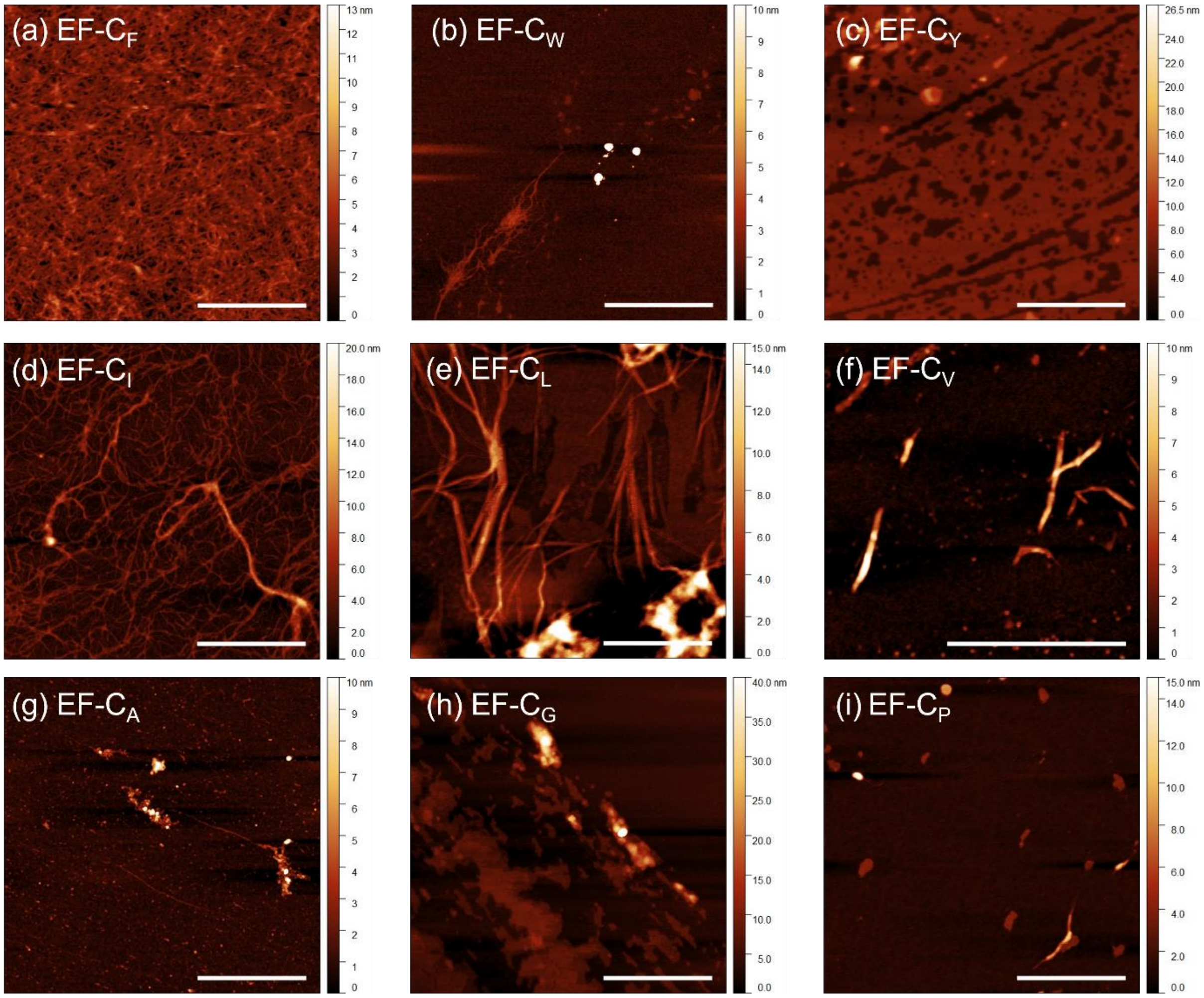

Figure S9. AFM overview images of the assembled morphologies of EF-C variants. (a) AFM image of EF-C$_F$. (b) AFM image of EF-C$_W$. (c) AFM image of EF-C$_Y$. (d) AFM image of EF-C$_I$. (e) AFM image of EF-C$_L$. (f) AFM image of EF-C$_V$. (g) AFM image of EF-C$_A$. (h) AFM image of EF-C$_G$. (i) AFM image of EF-C$_P$. Scale bars of 1 μm.

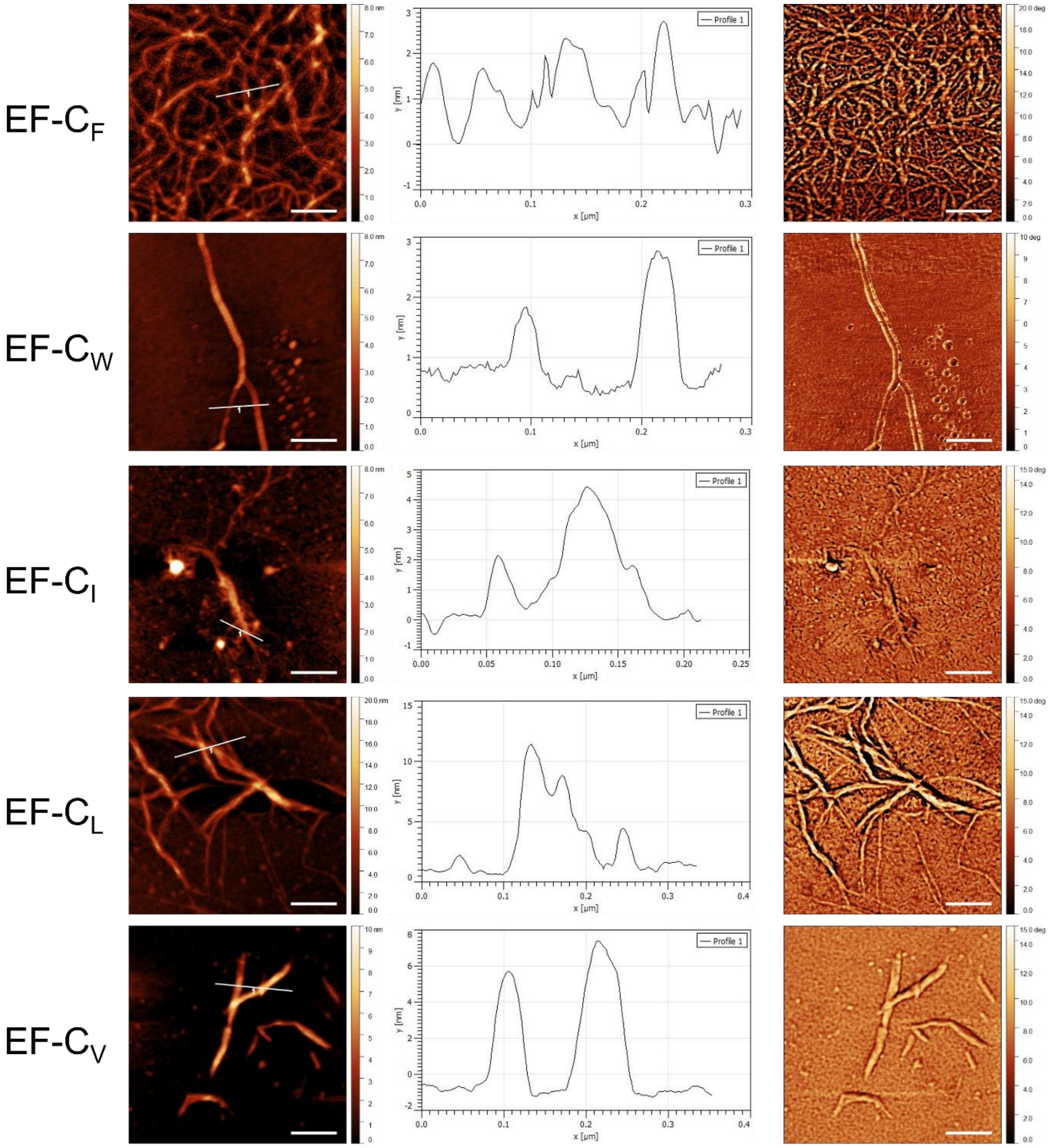

Figure S10. AFM detail images of the assembled morphologies of EF-C variants. (left) AFM height images of EF-C_F, EF-C_W, EF-C_I, EF-C_L, and EF-C_V. (center) Height profile of the selected fibrils from the AFM images of EF-C_F, EF-C_W, EF-C_I, EF-C_L, and EF-C_V. (right) AFM phase images of EF-C_F, EF-C_W, EF-C_I, EF-C_L, and EF-C_V. Scale bars of 200 nm.

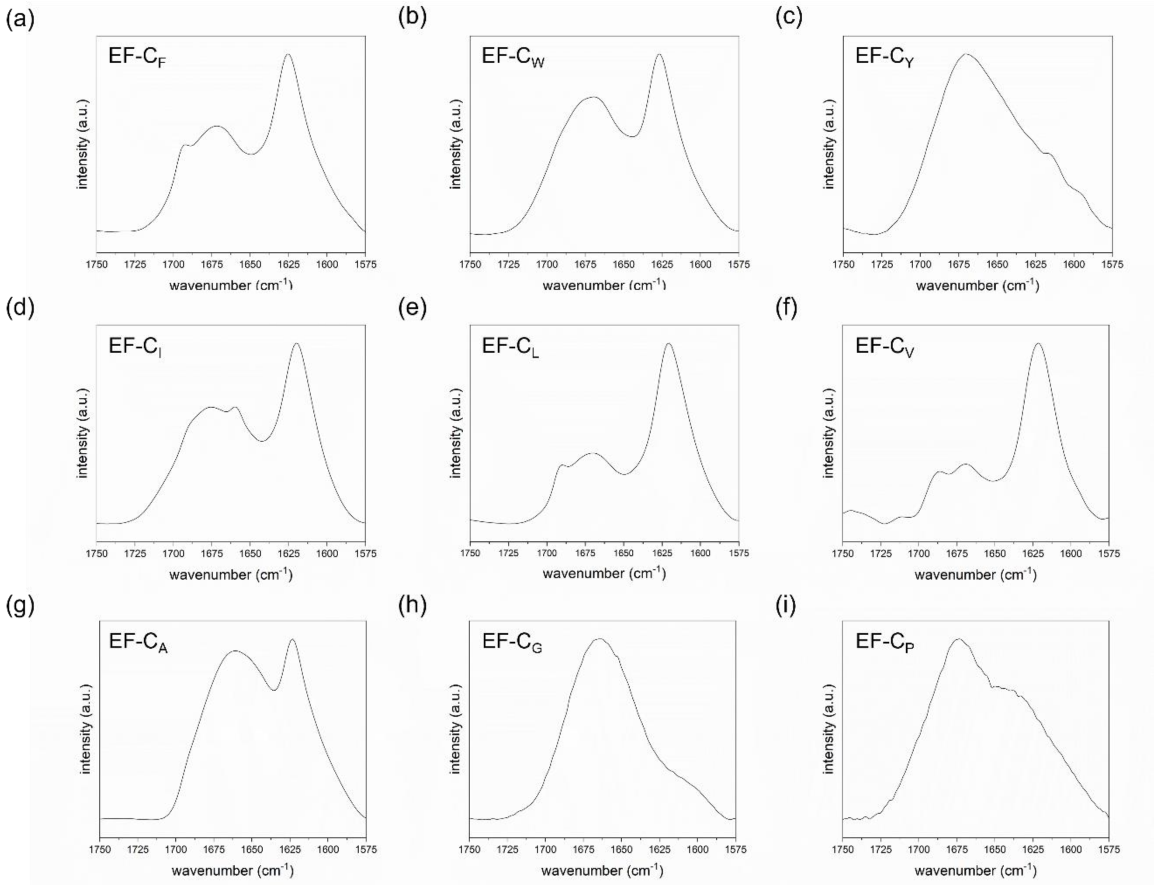

Figure S11. IR spectra of EF-C variants. (a) EF-C_F. (b) EF-C_W. (c) EF-C_Y. (d) EF-C_I. (e) EF-C_L. (f) EF-C_V. (g) EF-C_A. (h) EF-C_G. (i) EF-C_P.

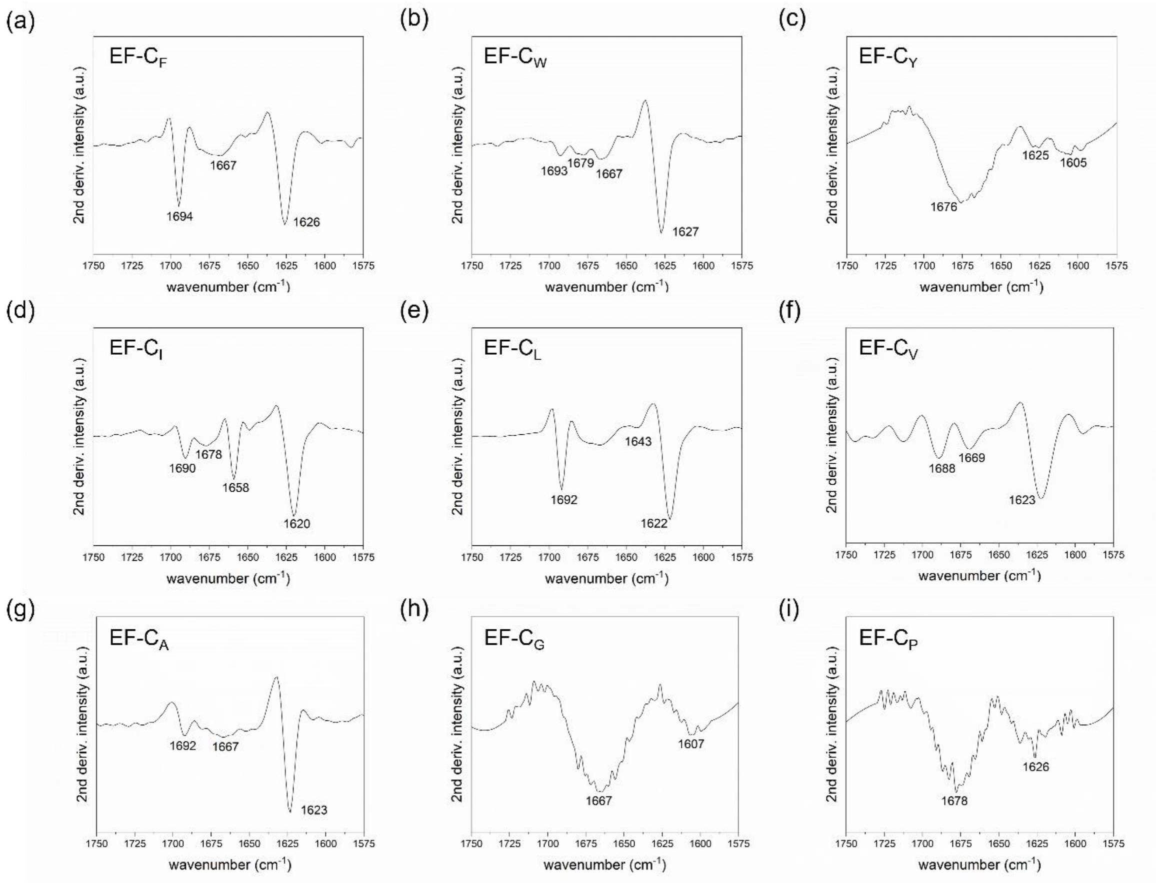

Figure S12. Second derivative IR spectra of EF-C variants. (a) EF-C$_F$. (b) EF-C$_W$. (c) EF-C$_Y$. (d) EF-C$_I$. (e) EF-C$_L$. (f) EF-C$_V$. (g) EF-C$_A$. (h) EF-C$_G$. (i) EF-C$_P$.

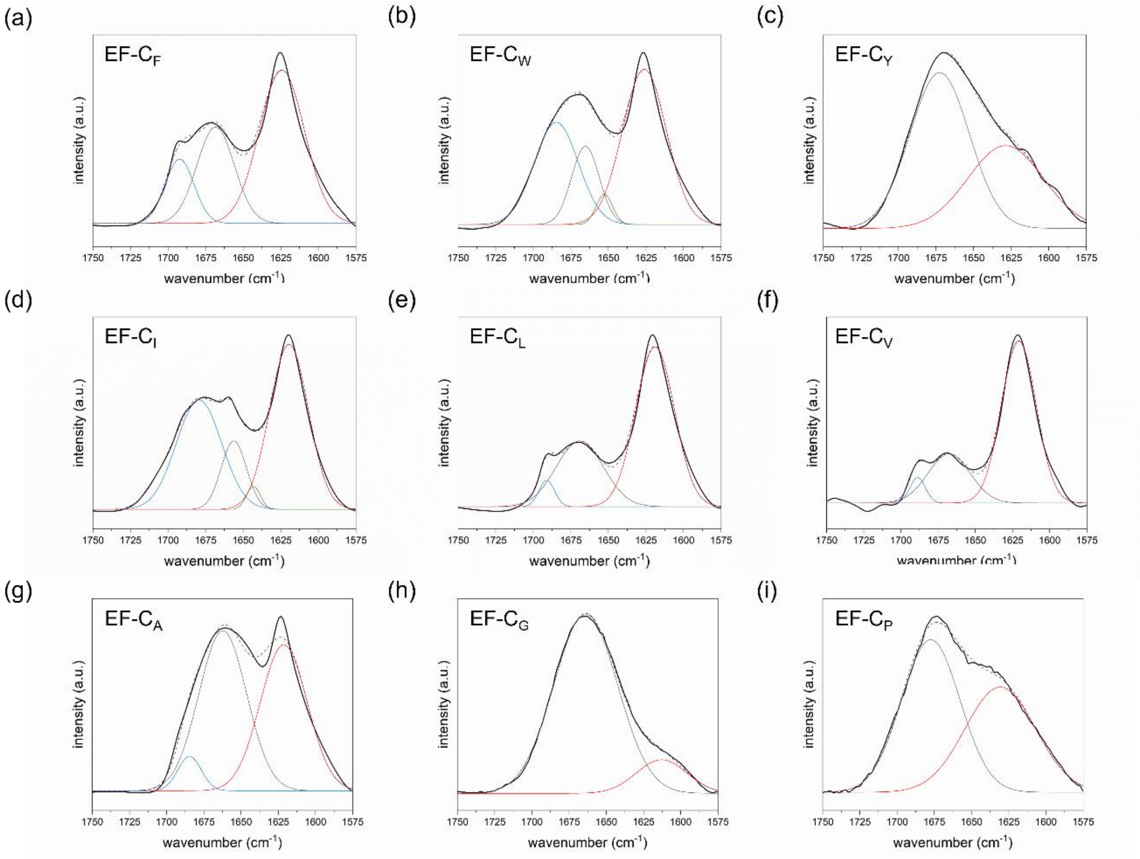

Figure S13. Deconvoluted IR spectra of EF-C variants. Black lines are the IR spectra, and the brown dotted line are the deconvoluted signals according to the 2nd derivative IR spectra. Red lines represent the signal contributed from parallel β-sheets (1615-1643 cm^{-1}), green lines show the signal derived from random coil and α-helix (1640-1670 cm^{-1}), gray lines display the signal stemming from β-turn (1665-1685 cm^{-1}), and blue lines exhibit the signal originating from anti- parallel β-sheets (1675-1700 cm^{-1}). (a) Deconvoluted IR spectrum of EF-C_F with relative β-sheets of 71.0%. (b) Deconvoluted IR spectrum of EF-C_W with relative β-sheets of 80.8%. (c) Deconvoluted IR spectrum of EF-C_Y with relative β-sheets of 40.3%. (d) Deconvoluted IR spectrum of EF-C_I with relative β-sheets of 84.7%. (e) Deconvoluted IR spectrum of EF-C_L with relative β-sheets of 68.0%. (f) Deconvoluted IR spectrum of EF-C_V with relative β-sheets of 74.2% (g) Deconvoluted IR spectrum of EF-C_A with relative β-sheets of 50.1%. (h) Deconvoluted IR spectrum of EF-C_G with relative β-sheets of 13.1%. (i) Deconvoluted IR spectrum of EF-C_P with relative β-sheets of 45.3%.

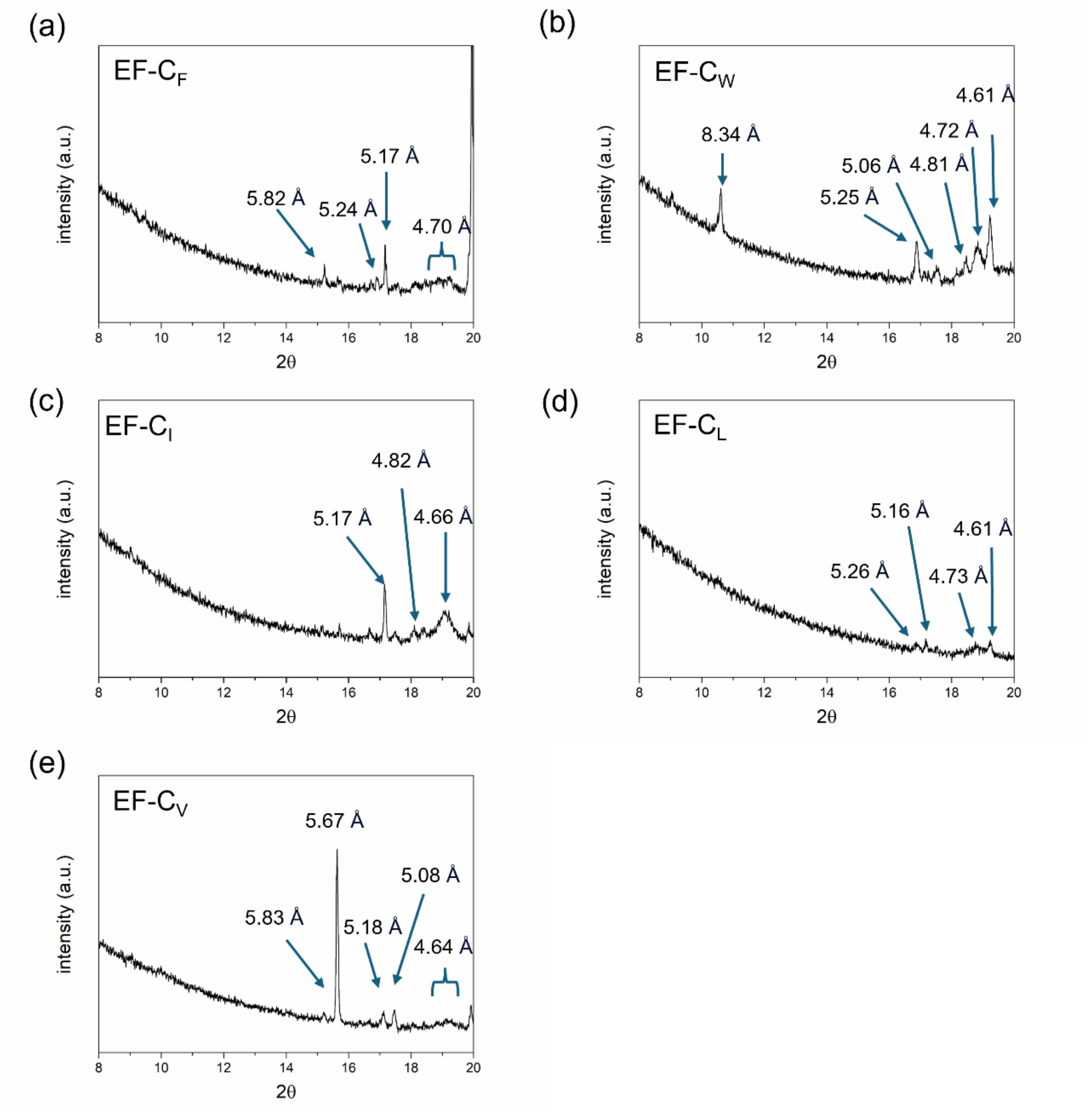

Figure S14. XRD diffractograms of self-assembled EF-C variants with assigned molecular packaging distances. (a) The XRD diffractogram of EF-C_F and the correlated molecular packing distances at 4.7, 5.17, 5.24, and 5.82 Å. (b) The XRD diffractogram of EF-C_W and the analogized molecular packing distances at 4.61, 4.72, 4.81, 5.06, 5.25, and 8.34 Å. (c) The XRD diffractogram of EF-C_I and the corresponding molecular packing distances at 4.66, 4.82, and 5.17 Å. (d) The XRD pattern of EF-C_L and the associated molecular packing distances at 4.61, 4.73, 5.16, and 5.26 Å. (e) The XRD pattern of EF-C_V and the relevant molecular packing distances at 4.64, 5.08, 5.18, 5.67, and 5.83 Å.

Table S1. Common secondary structures and frequencies (wavenumbers) of corresponding amide I bonds.

Secondary structure	Amide I frequencies (cm^{-1})
parallel β-sheets	1615-1643
random (unordered)	1640-1650
α-helix	1650-1670
β-turn	1665-1685
antiparallel β-sheets	1675-1700

Signals from 1600 cm^{-1} to 1620 cm^{-1} can be attributed to different side chains.[2–4]

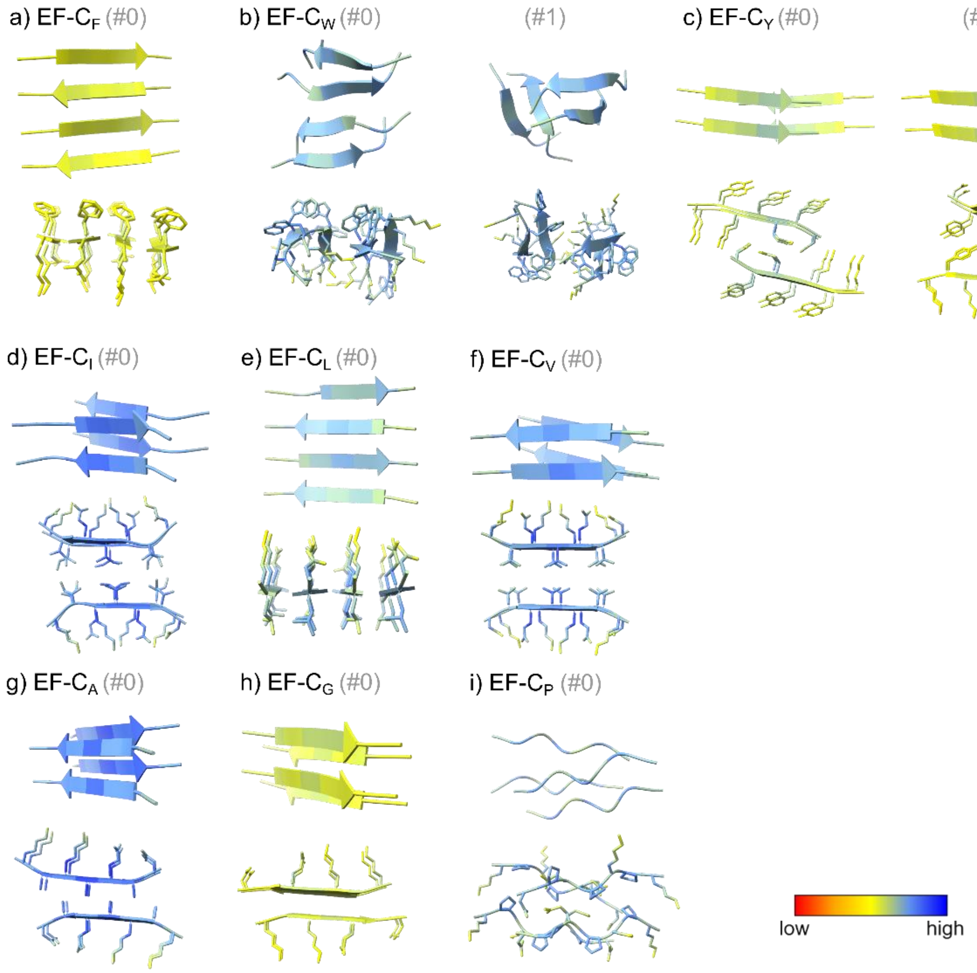

Figure S15. Structural models for peptide oligomers (4mers) of EF-C variants using AF3. Local confidence values (predicted local distance difference test, pLDDT) are used as color coding. Low confidence regions are shown in red/yellow, high confidence regions in blue. While the top model (highest confidence, #0) has been used for MD simulations by default, for some peptides, another model (e.g. #1, #4) with very similar confidence has been considered as this showed consistence in hydrophobic side chains to the core. The hydrophobic core of the fibrils was formed by the hydrophobic side chains, while charged residues were exposed to the outside. An exception is EF-C$_Y$ (c), for which AF3 predicted not only such an arrangement, but also an assembly with charged groups in the fibril core and Y residues exposed to the outside, most likely due to the polar -OH groups.

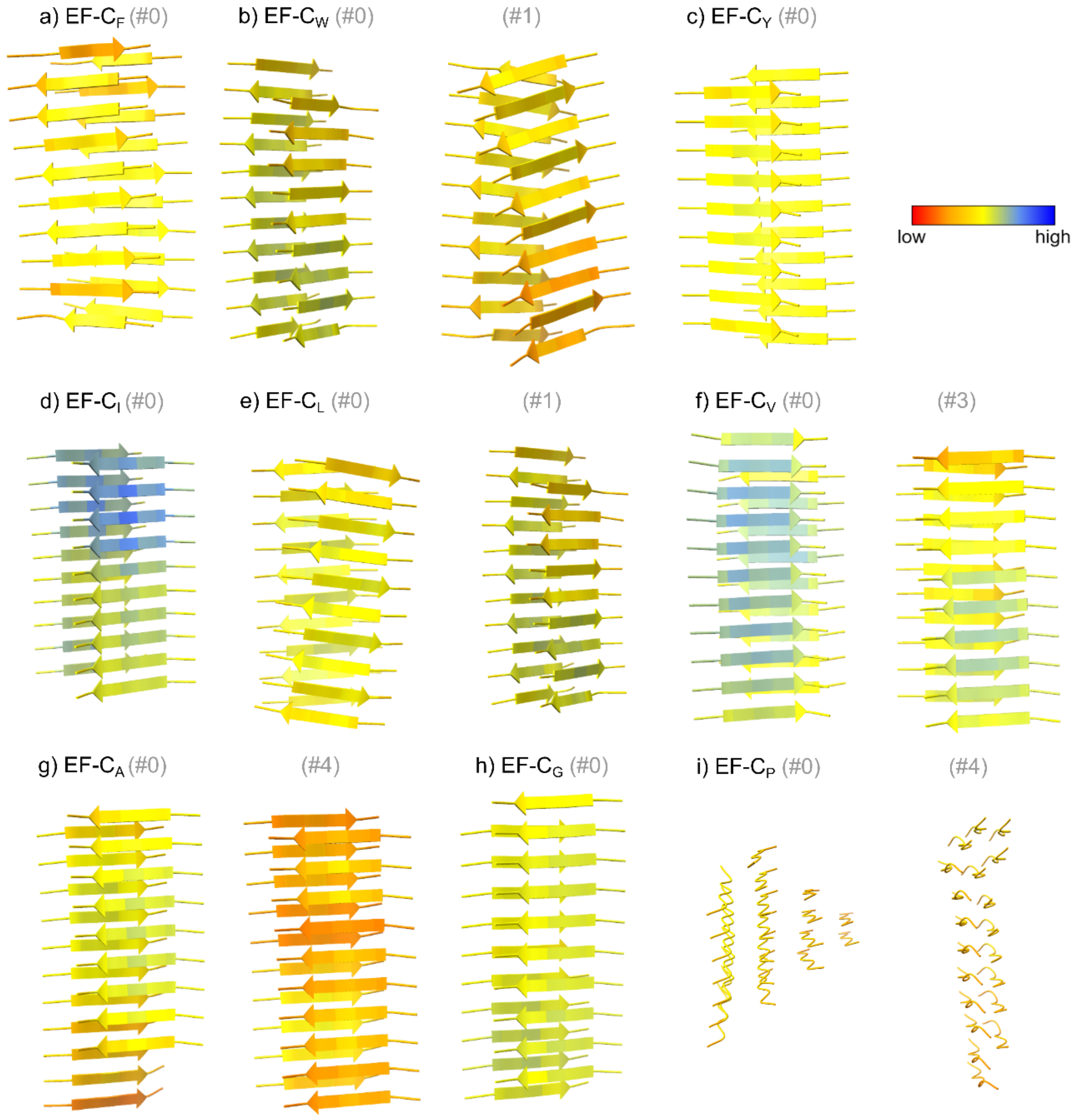

Figure S16. Structural models for peptide protofibrils (20mers) of EF-C variants using AF3. Local confidence values (predicted local distance difference test, pLDDT) are used as color coding. Low confidence regions are shown in red/yellow, high confidence regions in blue. While the top model (highest confidence, #0) has been used for MD simulations by default, for some peptides, another model (e.g. #1, #3, #4) with very similar confidence has been considered as this showed consistence in two-stranded sheets or twisting. For the 20mers, all peptide variants were characterized by hydrophobic cores formed by the hydrophobic amino acids which we varied in this study.

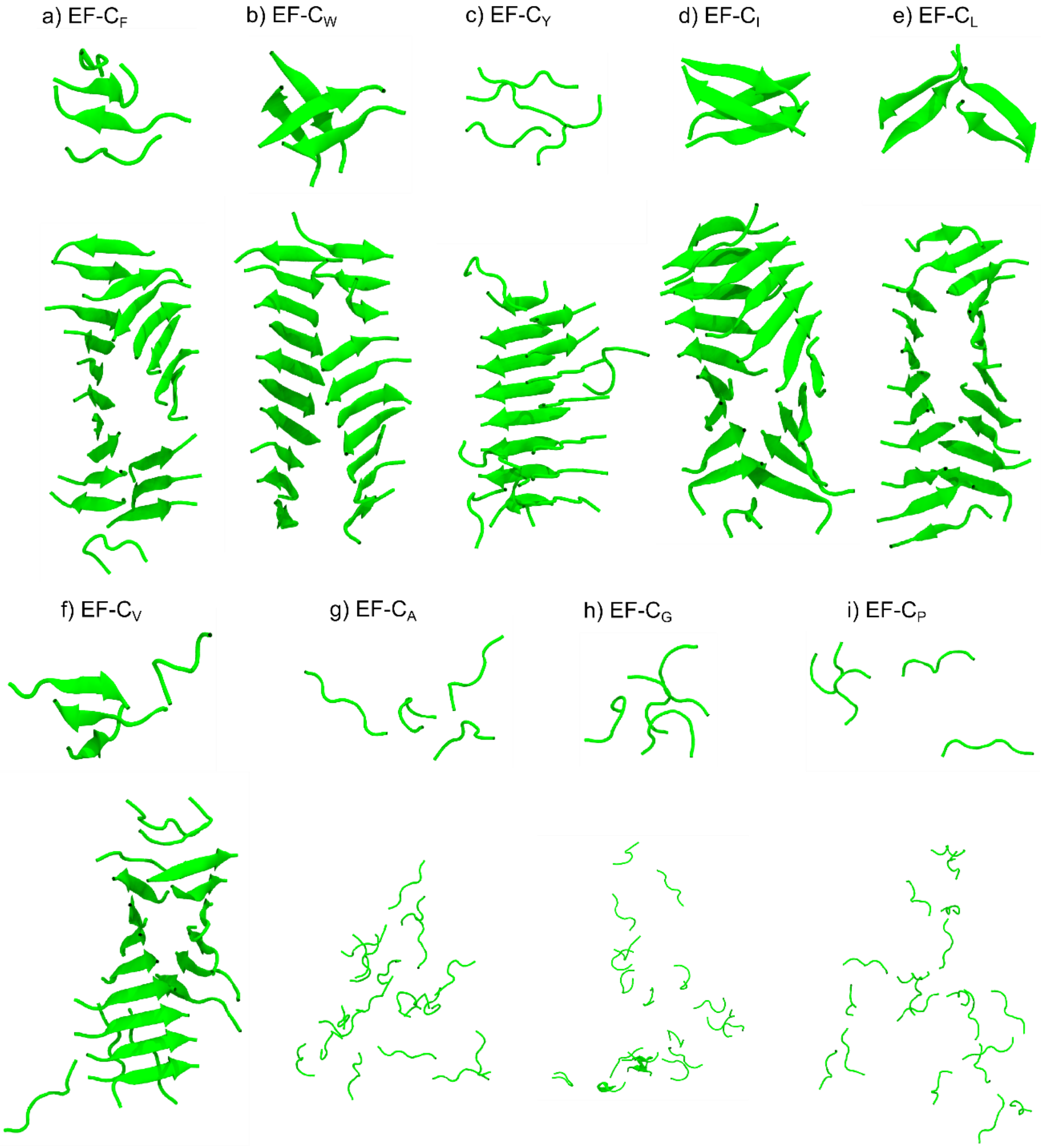

Figure S17. Representative structures of peptide oligomers (4mers) and protofibrils (20mers) of EF-C variants. Cartoons represent the central structures of the largest cluster of structures at the end of the simulations (determined from the final 10 ns of MD simulation trajectories of three replicates each). Clustering was performed using the gromos method with RMSD cutoff of 0.8 nm in GROMACS. Structures either disassembled during MD, representing unstable AF3 starting models, or stabilized their structures by twisting.

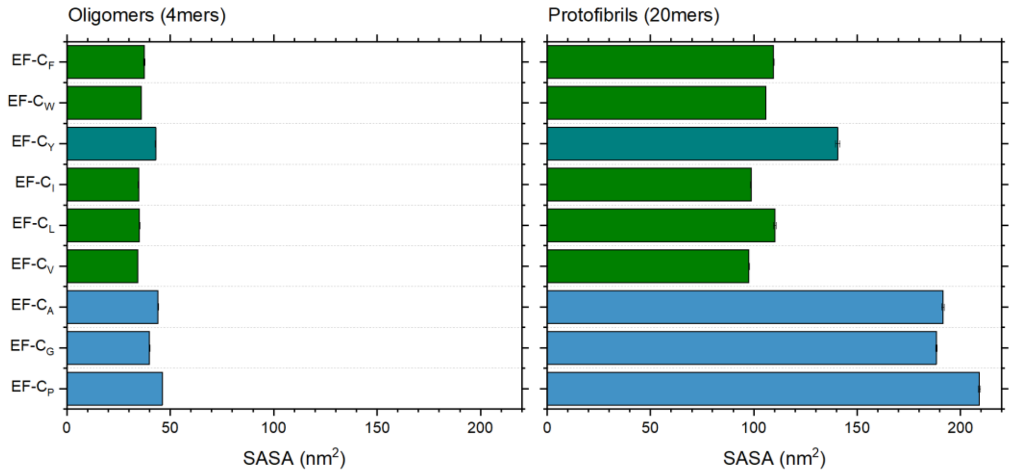

Figure S18. Solvent Accessible Surface Areas (SASAs) of EF-C oligomers (4mer) and protofibrils (20mers). The SASA of a tightly packed self-assembled oligomer or protofibril is smaller than the SASA of a loosely packed structure or unclustered peptide monomers. Means with standard errors are shown.

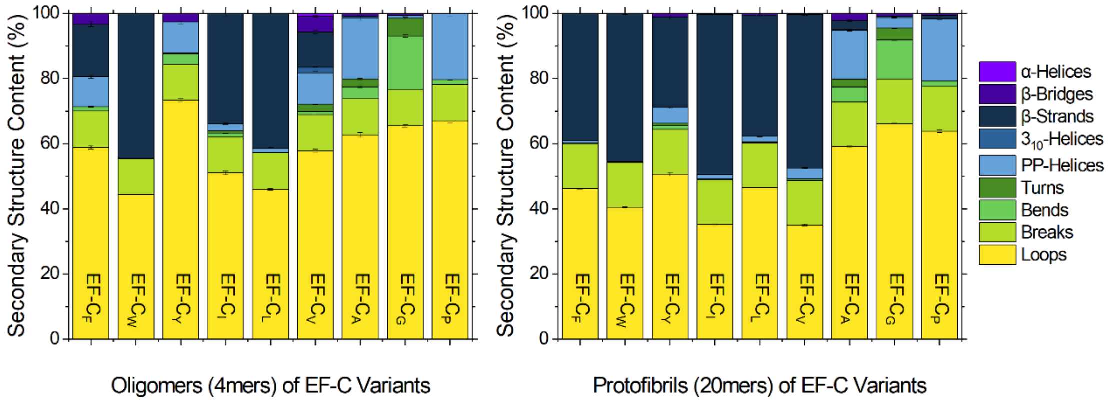

Figure S19. Secondary structure content of EF-C oligomers (4mer) and protofibrils (20mers). The average secondary structures (and standard errors) were determined for the last 10 ns simulation time of all replicates.

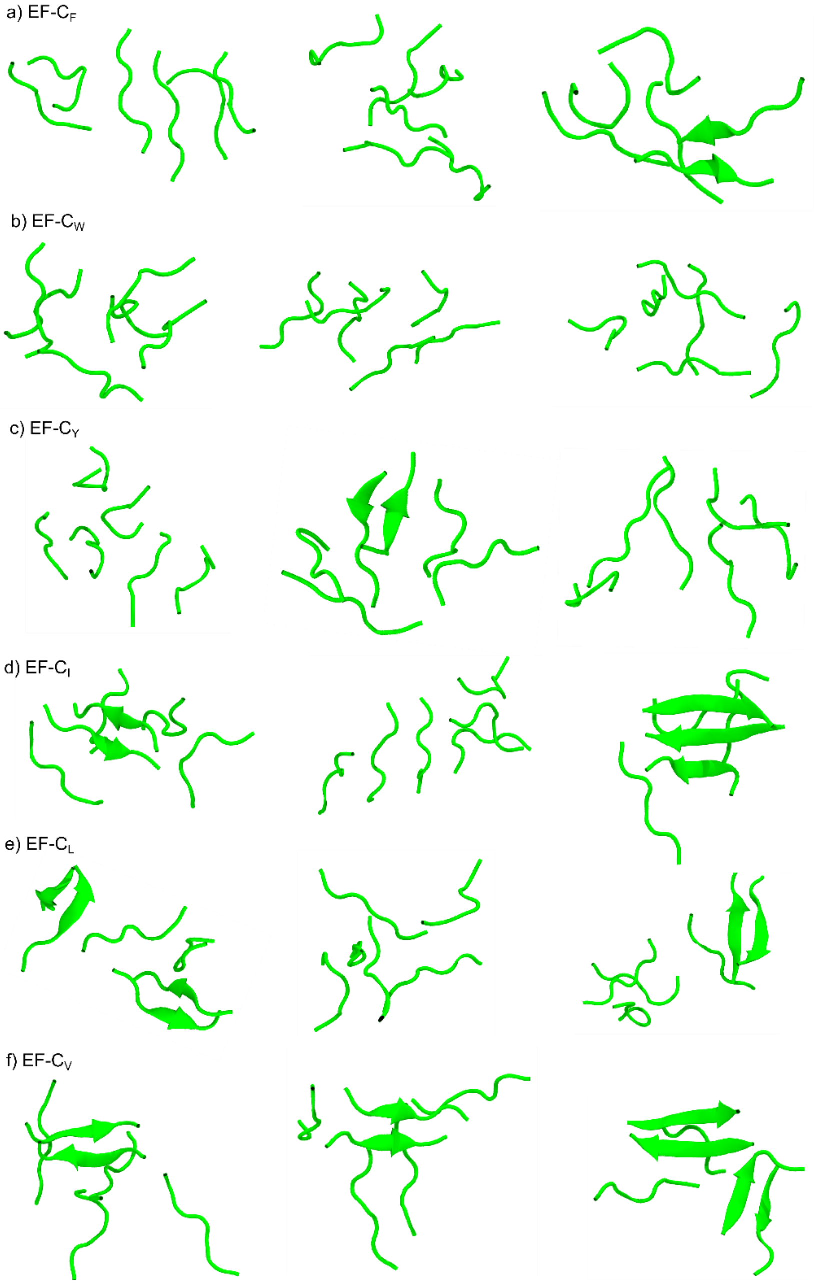

Figure S20. Self-assembled peptide structures of fibril-forming EF-C variants. Structures represent the central structures of the three largest clusters of the self-assembly of six monomers during the final 10 ns of MD simulations of three replicates each. Clustering was performed using the gromos method with RMSD cutoff of 0.8 nm in GROMACS. Formation of dimers and trimers indicates early oligomers while not all peptides transformed their secondary structure within the simulation time such as EF-C_W.

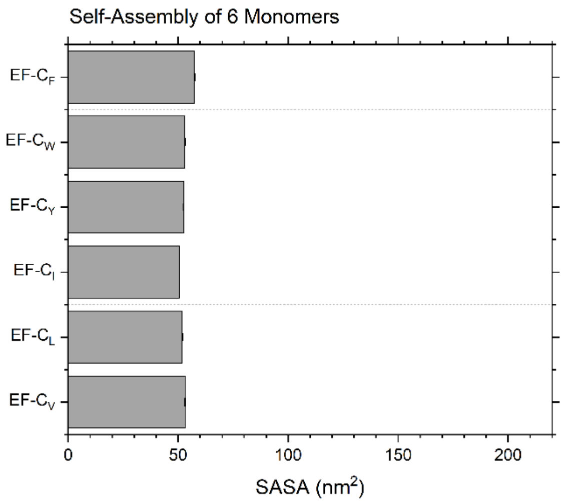

Figure S21. Solvent Accessible Surface Areas (SASAs) of EF-C self-assembled structures with six monomers. All fibril-forming EF-C variants clustered during self-assembly and reached similar SASAs within 500 ns simulation time. Means with standard errors are shown.

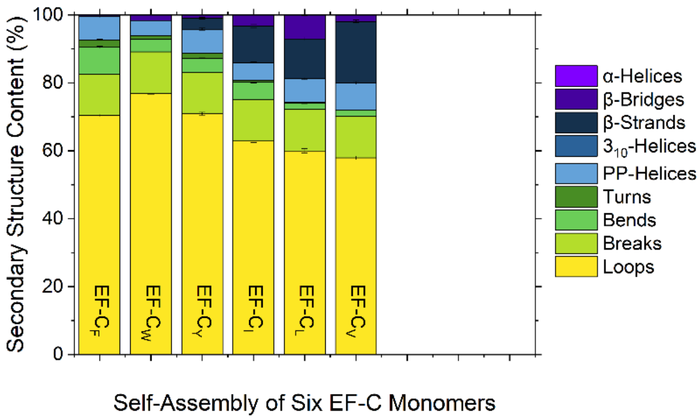

Figure S22. Secondary structure content of EF-C self-assembled structures with six monomers. The average secondary structures (and standard errors) were determined for the last 10 ns simulation time of all replicates.

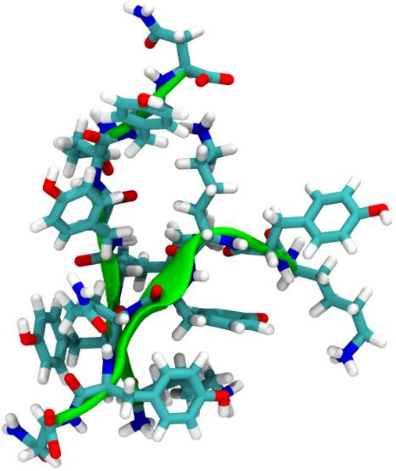

Figure S23. Structural representation of dimer of EF-C$_Y$ (KYKYQYN) formed in simulation. Tyrosine and lysine residues are both directed outwards with proximity between cationic lysine amine groups and aromatic tyrosine residues.

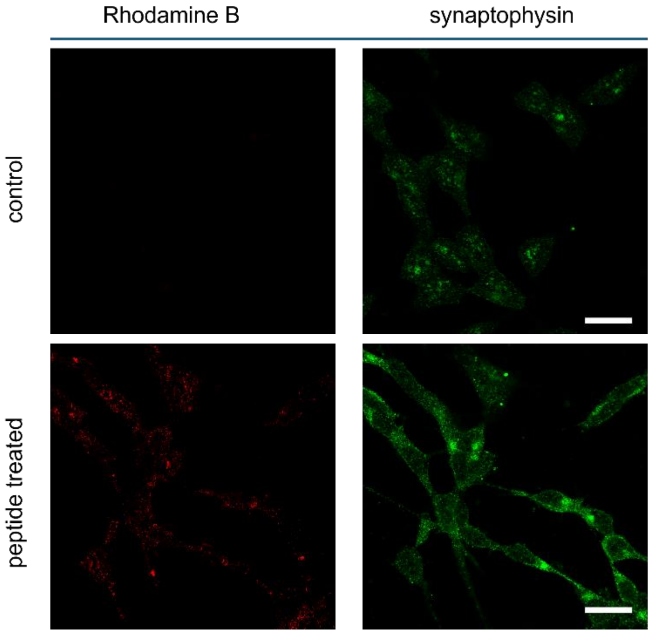

Figure S24. Raw data of fluorescence images of immunostaining in Figure 5 (f) (scale bar = 20 μm).

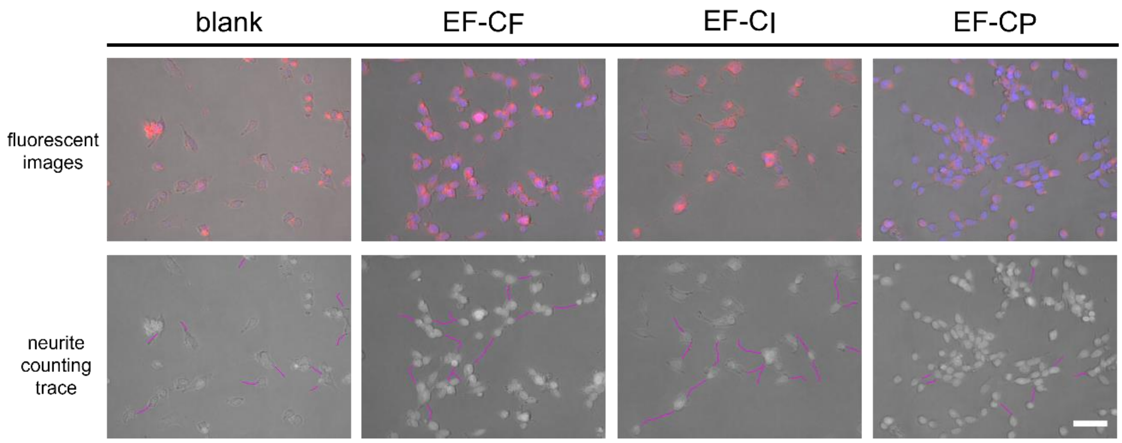

Figure S25. Examples of neurite counting traces. The upper panels show the fluorescent images of SH-SY5Y cells with stained nuclei (Hoechst 33342) and cell membranes (Deep Red Plasma Membrane). Based on the fluorescent images above, neurites were marked in pink, measured, and calculated from the images in the lower panels based on previous reports (scale bar = 50 μm).[5,6]

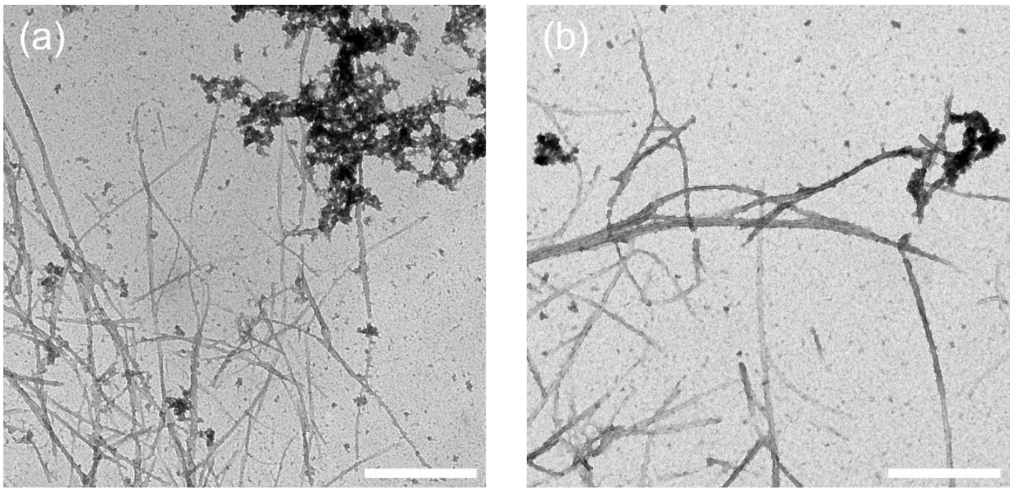

Figure S26. TEM images of co-assembled EF-C$_{I}$ and RhB-EF-C$_{I}$ (v/v= 9:1). (a) TEM image with a scale bar of 500 nm. (b) TEM image with a scale bar of 250 nm.

Supporting References

(1) Birtalan, E.; Rudat, B.; Kölmel, D. K.; Fritz, D.; Vollrath, S. B. L.; Schepers, U.; Bräse, S. Investigating Rhodamine B-Labeled Peptoids: Scopes and Limitations of Its Applications. *Biopolymers* **2011**, *96* (5), 694–701. https://doi.org/10.1002/bip.21617.

(2) Calero, M.; Gasset, M. Featuring Amyloids with Fourier Transform Infrared and Circular Dichroism Spectroscopies. In *Amyloid Proteins: Methods and Protocols*; Sigurdsson, E. M., Calero, M., Gasset, M., Eds.; Humana Press: Totowa, NJ, 2012; pp 53–68. https://doi.org/10.1007/978-1-61779-551-0_5.

(3) Moran, A.; Mukamel, S. The Origin of Vibrational Mode Couplings in Various Secondary Structural Motifs of Polypeptides. *Proceedings of the National Academy of Sciences* **2004**, *101* (2), 506–510. https://doi.org/10.1073/pnas.2533089100.

(4) Seshadri, S.; Khurana, R.; Fink, A. L. [36] Fourier Transform Infrared Spectroscopy in Analysis of Protein Deposits. In *Methods in Enzymology*; Academic Press, 1999; Vol. 309, pp 559–576. https://doi.org/10.1016/S0076-6879(99)09038-2.

(5) Meijering, E.; Jacob, M.; Sarria, J.-C. F.; Steiner, P.; Hirling, H.; Unser, M. Design and Validation of a Tool for Neurite Tracing and Analysis in Fluorescence Microscopy Images. *Cytometry Part A* **2004**, *58A* (2), 167–176. https://doi.org/10.1002/cyto.a.20022.

(6) Meijering, E. Neuron Tracing in Perspective. *Cytometry Part A* **2010**, *77A* (7), 693–704. https://doi.org/10.1002/cyto.a.20895.
